# Forkhead box O3 protects the heart against paraquat‐induced aging‐associated phenotypes by upregulating the expression of antioxidant enzymes

**DOI:** 10.1111/acel.12990

**Published:** 2019-07-01

**Authors:** Zao‐Shang Chang, Jing‐Bo Xia, Hai‐Yan Wu, Wen‐Tao Peng, Fu‐Qing Jiang, Jing Li, Chi‐Qian Liang, Hui Zhao, Kyu‐Sang Park, Guo‐Hua Song, Soo‐Ki Kim, Ruijin Huang, Li Zheng, Dong‐Qing Cai, Xu‐Feng Qi

**Affiliations:** ^1^ Key Laboratory of Regenerative Medicine of Ministry of Education, Department of Developmental & Regenerative Biology Jinan University Guangzhou China; ^2^ Department of Surgery, Union Hospital, Tongji Medical College Huazhong University of Science and Technology Wuhan China; ^3^ Key Laboratory of Regenerative Medicine of Ministry of Education, School of Biomedical Sciences, Faculty of Medicine The Chinese University of Hong Kong Hong Kong SAR China; ^4^ Department of Physiology, Wonju College of Medicine Yonsei University Wonju Korea; ^5^ Institute of Atherosclerosis TaiShan Medical University Tai'an China; ^6^ Department of Microbiology Wonju College of Medicine, Yonsei University Wonju Korea; ^7^ Institute of Anatomy, Department of Neuroanatomy, Medical Faculty Bonn Rheinische Friedrich-Wilhelms-University of Bonn Bonn Germany; ^8^ School of Environmental Science and Engineering Guangdong University of Technology Guangzhou China

**Keywords:** aging, antioxidant enzyme, cardiac dysfunction, Forkhead box O3, paraquat

## Abstract

Paraquat (PQ) promotes cell senescence in brain tissue, which contributes to Parkinson's disease. Furthermore, PQ induces heart failure and oxidative damage, but it remains unknown whether and how PQ induces cardiac aging. Here, we demonstrate that PQ induces phenotypes associated with senescence of cardiomyocyte cell lines and results in cardiac aging‐associated phenotypes including cardiac remodeling and dysfunction in vivo. Moreover, PQ inhibits the activation of Forkhead box O3 (FoxO3), an important longevity factor, both in vitro and in vivo. We found that PQ‐induced senescence phenotypes, including proliferation inhibition, apoptosis, senescence‐associated β‐galactosidase activity, and p16^INK4a^ expression, were significantly enhanced by FoxO3 deficiency in cardiomyocytes. Notably, PQ‐induced cardiac remolding, apoptosis, oxidative damage, and p16^INK4a^ expression in hearts were exacerbated by FoxO3 deficiency. In addition, both in vitro deficiency and in vivo deficiency of FoxO3 greatly suppressed the activation of antioxidant enzymes including catalase (CAT) and superoxide dismutase 2 (SOD2) in the presence of PQ, which was accompanied by attenuation in cardiac function. The direct in vivo binding of FoxO3 to the promoters of the *Cat* and *Sod2* genes in the heart was verified by chromatin immunoprecipitation (ChIP). Functionally, overexpression of *Cat* or *Sod2* alleviated the PQ‐induced senescence phenotypes in FoxO3‐deficient cardiomyocyte cell lines. Overexpression of FoxO3 and CAT in hearts greatly suppressed the PQ‐induced heart injury and phenotypes associated with aging. Collectively, these results suggest that FoxO3 protects the heart against an aging‐associated decline in cardiac function in mice exposed to PQ, at least in part by upregulating the expression of antioxidant enzymes and suppressing oxidative stress.

## 1 INTRODUCTION

Oxidative stress is one of the most prominent hallmarks of aging and is characterized by the combination of higher reactive oxygen species (ROS) generation and impaired antioxidant defense (Kirkwood, 2005; Sohal & Weindruch, 1996). Increased oxidative stress induces a functional decline of cellular repair mechanisms and contributes to the onset and progression of age‐associated pathologies, including cardiovascular diseases (Finkel & Holbrook, 2000; Wang & Bennett, 2012). Increasing lines of evidence suggest that overexpression of antioxidant molecules, including catalase, thioredoxin, and coenzyme Q10, significantly induces lifespan extension (Schriner et al., 2005; Tarry‐Adkins et al., 2013). Moreover, mice with deficiency in mitochondrial electron transfer‐associated genes such as *mclk1* or *p66^shc^* have longer lifespans (Liu et al., 2005; Migliaccio et al., 1999). These studies suggest that oxidative stress plays an important role in aging‐related disease. Aging is the most important risk factor that increases the susceptibility to developing cardiovascular disease. The relevance of oxidative stress has been suggested for both heart aging and the development of cardiac diseases such as heart failure, cardiac hypertrophy, and diabetic cardiomyopathy (Griendling et al., 2016; Touyz, Anagnostopoulou, Lucca, & Montezano, 2016).

Paraquat (1, 1′‐dimethyl‐4,4′‐bipyridinium dichloride, PQ), a quaternary nitrogen herbicide without selection, is widely used in worldwide agricultural practices. However, PQ is highly toxic to humans and associated with high mortality because of no effective treatment. Long time exposure to PQ is a risk factor for the age‐related neurodegenerative diseases such as Parkinson's disease (Pezzoli & Cereda, 2013). Moreover, PQ exposure can result in failure and damage in multiple organs including the lung, heart, liver, and kidney (Wang, Zhu, Xiong, & Ren, 2017). In particular, PQ‐induced oxidative stress suppresses myocardial survival, impairs myocardial contractive function, and promotes heart failure (Ge, Zhang, Han, & Ren, 2010; Vinciguerra et al., 2012). Thus, PQ has been generally accepted to produce experimental models of oxidative stress or injury in multiple organs including the heart. PQ has been demonstrated to induce astrocytic senescence and senescence‐associated secretory phenotype (SASP) production in vitro and in vivo (Chinta et al., 2018). These previous studies have indicated that PQ might be a suitable factor for inducing experimental models of oxidative injury and aging.

Forkhead box O3 (FoxO3) is a member of the ubiquitously expressed FoxO proteins that also includes FoxO1, FoxO4, and FoxO6 in mammals. FoxO proteins are an evolutionarily conserved family of transcription factors that regulate a variety of biological processes, including the oxidative stress response, aging, metabolism, apoptosis, autophagy, and immunity (Martins, Lithgow, & Link, 2016; Matsuzaki et al., 2018). In mammals, although four members have overlapping functions, each FoxO protein regulates distinct gene expression programs in a tissue‐specific manner (Hosaka et al., 2004; Matsuzaki et al., 2018). In the heart, both FoxO1 and FoxO3 are highly expressed (Hannenhalli et al., 2006). Combined deletion of FoxO1 and FoxO3 specifically in cardiomyocytes results in increased cardiac injury following acute ischemia and reperfusion in vivo, suggesting that FoxO1 and FoxO3 protect the heart from oxidative stress (Sengupta, Molkentin, Paik, DePinho, & Yutzey, 2011). Mice lacking FoxO1 are embryonically lethal by E10.5 due to impaired vasculogenesis, and mice lacking FoxO3 are viable but develop cardiac hypertrophy as adults (Hosaka et al., 2004; Ni et al., 2006), indicating the different functions of FoxO1 and FoxO3 in the cardiovascular system. In the cardiovascular system, existing data of FoxO3 are controversial and suggest a complex function for FoxO3. In cardiac microvascular endothelial cells (CMECs), FoxO3 contributes to hypoxia‐induced ROS accumulation and apoptosis (Zhang et al., 2013). However, overexpression of FoxO3 in primary CMECs suppresses senescence and ROS accumulation by increasing the activity of antioxidants including catalase and SOD (Qi et al., 2015). For cardiomyocytes, a recent study reported that FoxO3 triggers cardiomyocyte apoptosis under hyperglycemic ischemia condition (Chen et al., 2018). In contrast, FoxO3 knockdown sensitizes cardiomyocytes to apoptosis, whereas overexpression of FoxO3 suppresses apoptosis (Lu et al., 2013). Moreover, FoxO3 knockdown in cardiomyocytes drastically blocked apelin‐mediated anti‐apoptotic activity in response to hypoxia (Boal et al., 2015). These reports suggest that the role of FoxO3 in cardiac injury is complex and variable under different conditions.

Additionally, in vitro and in vivo studies have demonstrated FoxO3 as a longevity factor that plays an important role in lifespan extension in different organisms, including humans (Flachsbart et al., 2017; Martins et al., 2016; Wessells, Fitzgerald, Cypser, Tatar, & Bodmer, 2004). These previous studies have strongly proposed that the longevity factor FoxO3 might be a suitable target for preventing the aging process of organs and organisms. However, the specific functions of FoxO3 involved in protection from PQ‐induced cardiac injury and aging are largely unknown. In the present study, we report that PQ exposure results in oxidative stress in cultured cardiomyocyte cell lines and hearts, and this is accompanied by phenotypes associated with senescence and aging, including proliferation arrest, apoptosis, cardiac remodeling, and dysfunction, as well as p16^INK4a^ elevation. Significantly, FoxO3 deficiency further exacerbates PQ‐induced phenotypes associated with in vitro cardiomyocyte cell line senescence and in vivo cardiac aging by directly upregulating catalase (CAT) and superoxide dismutase 2 (SOD2) and suppressing oxidative stress. Our data suggest that strategies aimed at targeting FoxO3 hold promise as potential therapies for mitigating the development of phenotypes associated with cardiac aging.

## 2 RESULTS

### 2.1 Exposure of H9c2 cells to PQ induces senescence

**Figure 1:**
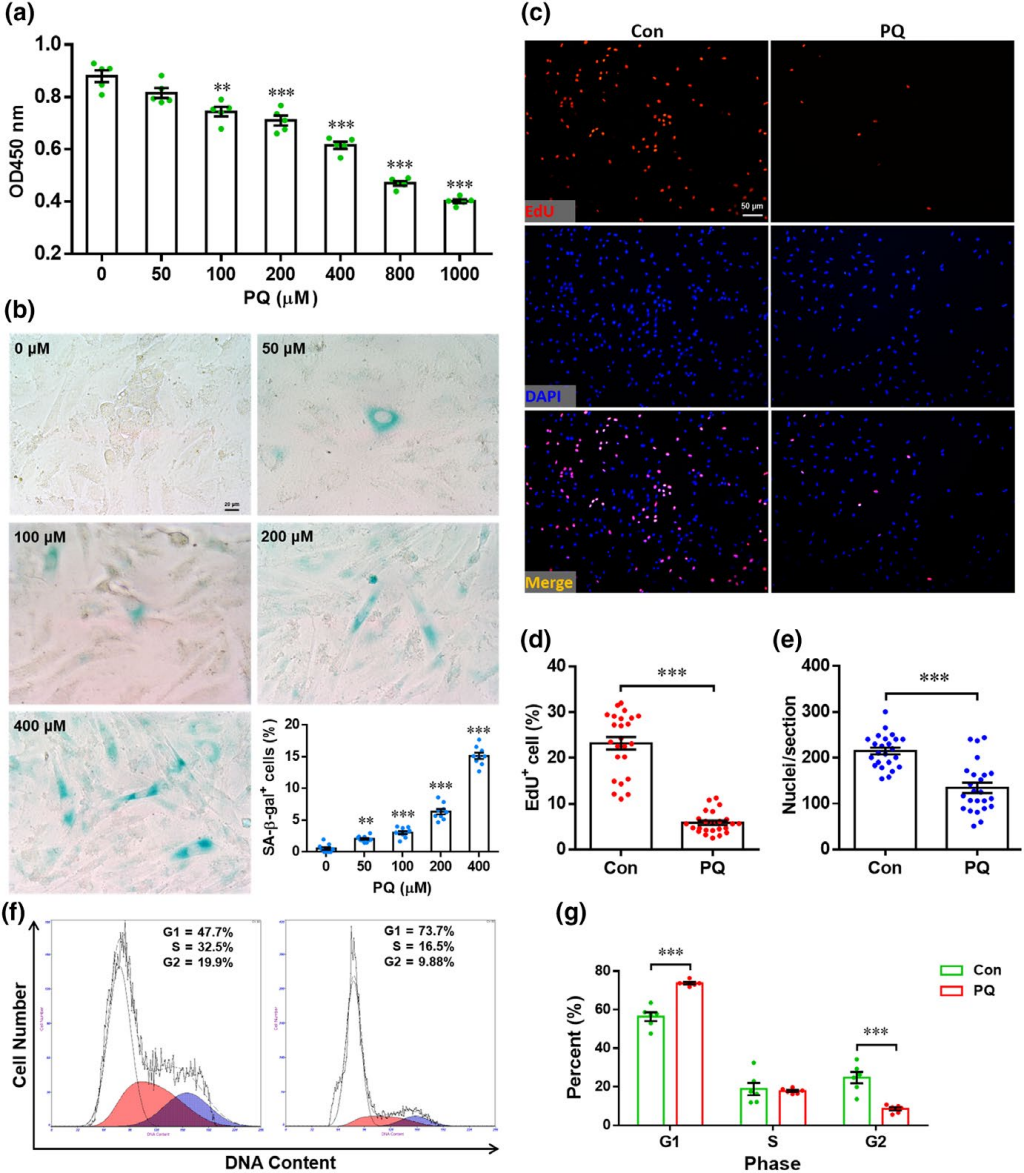
Paraquat (PQ) induces senescence of H9c2 cells. (a) H9c2 cells were incubated with PQ (0–1,000 μM) for 24 hr. The cell survival activity was examined by CCK8 Cell Counting Kit and expressed as the absorbance at 450 nm. Results are presented as mean ± *SEM* (*n* = 5), **p < 0.01, ***p < 0.001 versus control group. (b) H9c2 cells were incubated with PQ (0–400 μM) for 4 hr/day. Three days later, cell senescence is analyzed by senescence‐associated β‐galactosidase (SA‐β‐Gal) staining. Representative images for each condition and quantification of SA‐β‐Gal^+^ cells are shown. Results are presented as mean ± *SEM* (*n* = 9 experiments), ***p < 0.001. (c–e) H9c2 cells were incubated with PQ (400 μM) for 4 hr, followed by proliferation evaluation by 5‐ethynyl‐2′‐deoxyuridine (EdU) labeling. (c) The representative images for each condition are shown. Quantification of EdU^+^ cells (d) and nuclei (e) is shown. Results are presented as mean ± *SEM* (*n* = 24 sections from six experiments), ***p < 0.001. (f and g) H9c2 cells were incubated with PQ (400 μM) for 4 hr, followed by cell cycle assay to analyze proliferation. Representative images (f) and quantification (g) are shown. Results are presented as mean ± *SEM* (*n* = 6 experiments), ***p < 0.001

Paraquat‐induced oxidative stress impairs myocardial contraction and survival (Ge et al., 2010; Wang et al., 2017), prompting us to ask whether PQ exposure also suppresses the growth of cardiomyocytes. H9c2 cells, the rat cardiomyocyte cell line, were incubated with PQ (0–1 mM) for 24 hr; cell viability was then determined. As shown in Figure 1a, the viability of H9c2 cells was significantly suppressed by PQ exposure in a dose‐dependent manner. It has been demonstrated that PQ is capable of inducing the senescence of human fibroblasts and cultured astrocytes (Chinta et al., 2018; Jung, Hohn, Catalgol, & Grune, 2009). Therefore, we also asked whether PQ induces senescence in cardiomyocytes. H9c2 cells were treated with PQ (0–400 μM) for 3 days (4 hr/day), followed by the senescence‐associated β‐galactosidase (SA‐β‐Gal) staining. Even cells were continuously treatment for 24 hr by PQ, the IC50 is more than 1 mM (data not shown). Thus, it is likely safe for cells treated with PQ at 400 μM only for 4 hr. PQ exposure (4 hr/day for 3 days) induced a representative senescence phenotype in H9c2 cells (Figure 1b). The percentage of SA‐β‐Gal^+^cells was significantly increased by PQ in a dose‐dependent manner, with a highest induction of senescence at 400 μM. Thus, 400 μM of PQ was used to treat cells for 4 hr to examine cell biological parameters in below experiments.

To explore whether PQ exposure inhibits the proliferation of H9c2 cells, the nuclear incorporation of 5‐ethynyl‐2′‐deoxyuridine (EdU) was further examined. We found that the percentage of EdU^+^ cells was significantly decreased by PQ compared with PBS treatment alone (Figure 1c,d). Moreover, the nuclei density was also greatly decreased in PQ‐exposed cells (Figure 1e). In consistent, flow cytometry analysis revealed that PQ exposure induced cell cycle arrest in G1 phase (Figure 1f,g). These findings indicated that PQ exposure suppresses proliferation of H9c2 cells, which was accompanied by induction of senescence.

### 2.2 PQ inhibits activation of the Akt/FoxO3 pathway in H9c2 cells

We previously reported that FoxO3 activation is decreased in the senescent CMECs isolated from old rats, suggesting that the FoxO3 pathway may be an important mediator that regulates the senescence process (Qi et al., 2015). This finding promoted us to analyze FoxO3 activation in H9c2 cells exposed to PQ. The mRNA expression of FoxO3 was greatly decreased in H9c2 cells exposed to PQ (Figure S1A). Consistently, FoxO3 protein expression was also significantly inhibited by PQ exposure. However, the relative phosphorylation of FoxO3 at Ser 253 was significantly increased (Figure S1B–E). These data suggest that FoxO3 activation is suppressed by PQ exposure in H9c2 cells. It is well known that Akt is an upstream negative mediator of FoxO3 and promotes cell survival by phosphorylating and inhibiting the FoxO3 protein (Brunet et al., 1999). Under our experimental conditions, no significant difference in Akt expression was detected. However, Akt phosphorylation was remarkably suppressed by PQ exposure in H9c2 cells, suggesting inhibition of Akt activation (Figure S1B,F,G). These data indicate that Akt may not be necessary for the PQ‐mediated inactivation of FoxO3 in H9c2 cells.

### 2.3 Silencing of FoxO3 promotes PQ‐induced oxidative stress in H9c2 cells

**Figure 2:**
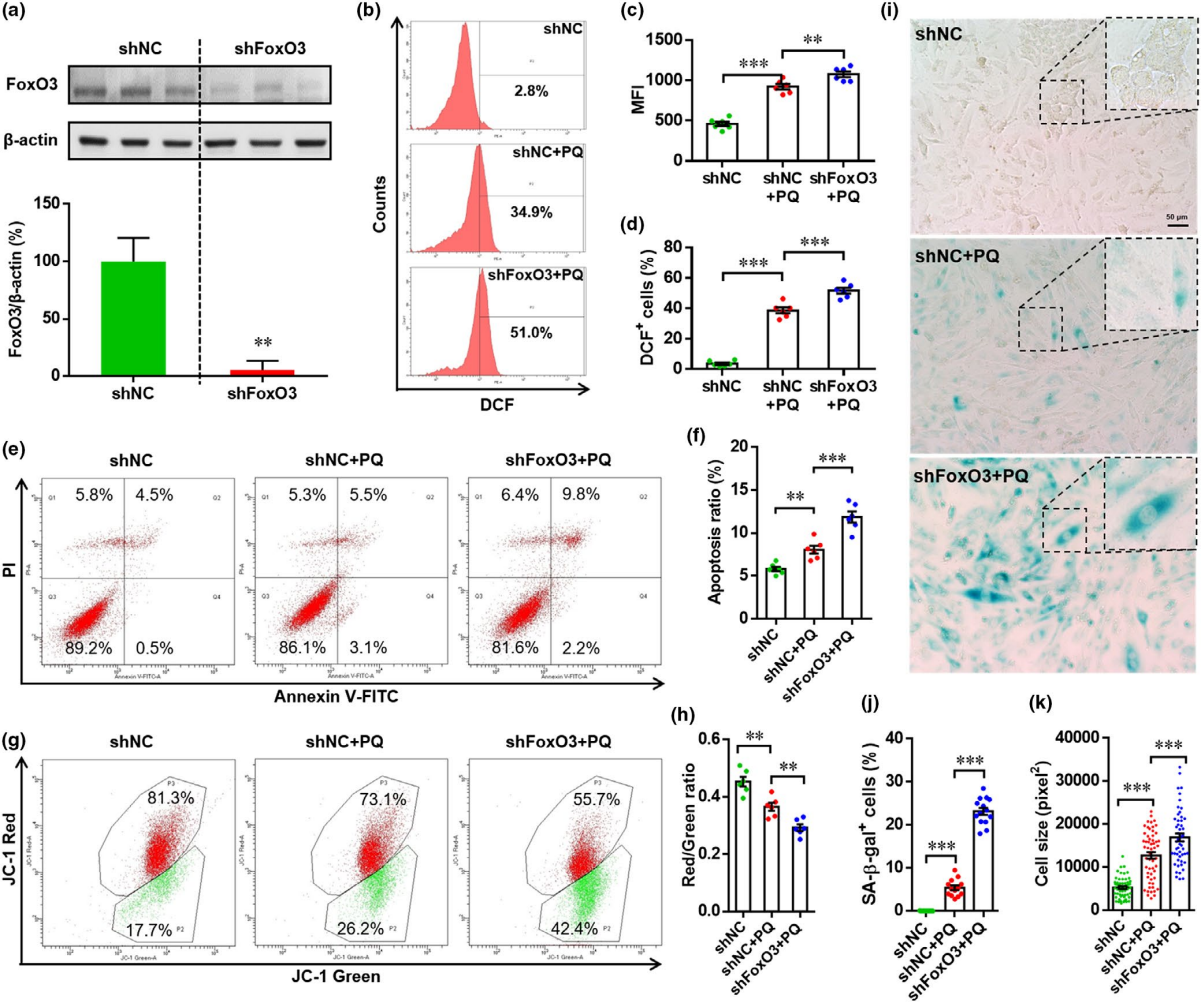
Forkhead box O3 (FoxO3) silencing promotes paraquat (PQ)‐induced reactive oxygen species (ROS) production and apoptosis in H9c2 cells. (a) H9c2 cells were transfected with lentivirus containing negative control shRNA or shRNA‐FoxO3 to establish stable cell lines by puromycin resistance. The FoxO3 knockdown efficiency was evaluated by Western blotting. Representative image (up panel) and relative quantification (down panel) were shown. Results are presented as mean ± *SEM* (*n* = 3), **p < 0.01 versus shNC. (b–d) Cells were incubated with or without PQ (400 μM) for 4 hr, and ROS generation was then evaluated by CM‐H_2_DCFDA staining and flow cytometry analysis. (b) Representative images of flow cytometry for each condition are shown. (c) Quantification of mean fluorescence intensity. Results are presented as mean ± *SEM* (*n* = 6), **p < 0.01, ***p < 0.001. (d) Quantification of DCF^+^ cells. Results are presented as mean ± *SEM* (*n* = 6), *p < 0.05, ***p < 0.001. (e and f) Cells were incubated with or without PQ (400 μM) for 4 hr, and apoptosis was then evaluated with Annexin V‐FITC/PI staining and flow cytometry analysis. (e) The representative images of flow cytometry are shown. (f) The percentage of apoptotic cells (both FITC^+^/PQI^−^ and FITC^+^/PI^+^) are statistically analyzed. Results are presented as mean ± *SEM* (*n* = 6), *p < 0.05. (g and h) Cells were incubated with or without PQ (400 μM) for 4 hr, and changes in mitochondrial membrane potential were then evaluated with JC‐1 staining and flow cytometry analysis. (g) Representative images are shown. (h) The fluorescence ratio of red to green is statistically analyzed. Results are presented as mean ± *SEM* (*n* = 6), *p < 0.05. (i–k) Cells are incubated with or without PQ (400 μM) as mentioned above; cell senescence is then analyzed by senescence‐associated β‐galactosidase (SA‐β‐Gal) staining. (i) The representative images for each condition are shown (scale bar = 50 μm). (j) SA‐β‐Gal^+^ cells are quantified as percentage of total cells. Results are presented as mean ± *SEM* (*n* = 12), ***p < 0.001. (k) Relative cell size is quantified as the mentioned in Methods section. Results are presented as mean ± *SEM* (about 100 cells are evaluated), ***p < 0.001

To further explore the potential protection of FoxO3 from PQ‐induced oxidative stress in H9c2 cells, a stable knockdown cell line was established using lentiviral‐mediated expression of an shRNA directed against FoxO3 (shFoxO3). Knockdown efficiency was examined by Western blotting analysis (Figure 2a). As shown in Figure 2b, PQ greatly increased the number of DCF^+^ cells in shNC group, and this was further increased by the FoxO3 knockdown. The PQ‐increased mean fluorescence intensity (MFI) and DCF^+^ cell numbers in shNC cells were also elevated by FoxO3 knockdown (Figure 2c,d). Many studies have demonstrated that oxidative stress induces the apoptosis of cardiomyocytes (Boccalini, Sassoli, Formigli, Bani, & Nistri, 2015; Dong et al., 2016). Thus, H9c2 apoptosis was also analyzed in this study. As expected, PQ exposure significantly increased the apoptosis of shNC cells, but knockdown of FoxO3 further promoted PQ‐induced apoptosis (Figure 2e,f). To examine the involvement of mitochondrial dysfunction in the PQ‐induced apoptosis, the ΔΨm was measured by flow cytometry and JC‐1 staining. As shown in Figure 2g, the percentage of the cell population with low red fluorescence, which indicates apoptosis, increased from 17.7% in shNC cells to 26.2% in those exposed to PQ. Forkhead box O3 deficiency further increased apoptosis (up to 42.4%). Statistical analysis of data from six independent experiments demonstrated that PQ exposure significantly reduced ΔΨm in shNC cells as indicated by a decrease in the red/green ratio from 0.42 ± 0.01 to 0.33 ± 0.01. Similarly, FoxO3 deficiency further significantly reduced the ΔΨm of PQ‐exposed cells (Figure 2h). We also explored the potential involvement of FoxO3 in PQ exposure‐induced senescence using the stable knockdown cell line. We found that PQ induced senescence in the shNC cells, and this was further increased by FoxO3 deficiency as indicated by the elevated percentage of SA‐β‐Gal^+^ cells and relative cell size (Figure 2i–k). Taken together, these data indicate that PQ exposure induces oxidative stress and apoptosis in H9c2 cells, which is further exacerbated by FoxO3 deficiency.

To further explore the mechanism by which FoxO3 protects against PQ‐induced oxidative injury in H9c2 cells, the expression of proteins associated with anti‐oxidation, apoptosis, and senescence was examined by Western blotting. We found that the expression of Cat and Sod2 was increased in shNC cells exposed to PQ compared with untreated cells; however, FoxO3 deficiency remarkably inhibited the PQ‐induced expression (Figure S2A–C), suggesting that FoxO3 may protect H9c2 cells from PQ‐mediated oxidative stress by regulating the expression of anti‐oxidases including CAT and SOD2. These results are consistent with previous studies in CMECs (Qi et al., 2015) and heart tissues (Sengupta et al., 2011). The Bax and Bcl2 expression ratio was increased in shNC cells treated with PQ, and this was further elevated by FoxO3 deficiency, indicating the anti‐apoptotic effects of FoxO3 (Figure S2A,D–F). Moreover, the FoxO3 deficiency induced a DNA damage response, which was evident by the activation of p53 (Figure S2A,G). Forkhead box O3 deficiency also increased the protein level of p16^INK4a^, a hallmark of senescence, in H9c2 cells treated with PQ (Figure S2A,H). These data are consistent with SA‐β‐Gal staining analysis results (Figure 2i–k).

### 2.4 PQ exposure suppresses the activation of FoxO3 in heart tissue

**Figure 3:**
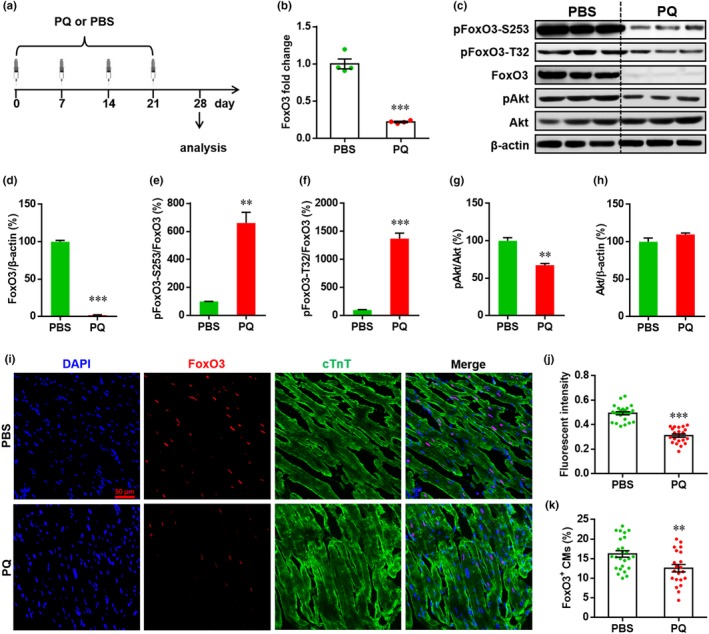
Paraquat (PQ) administration inhibits the activation of Forkhead box O3 (FoxO3) in heart. (a) Schematic of PQ injection experiments. PQ is administrated to mice (5 mg/Kg body) once per week for total 4 weeks. One week later after the last injection, heart samples are prepared and subjected to qRT–PCR, Western blotting and immunofluorescence staining analysis. PBS is used as negative control. (b) The relative expression of FoxO3 mRNA is shown. Results are presented as mean ± *SEM* (*n* = 4 hearts), ***p < 0.001. (c) Representative images of Western blotting for pan and phosphorylated FoxO3 and Akt. (d–h) The relative expression levels of target proteins are quantified as the percentage of control. Results are presented as mean ± *SEM* (*n* = 3 hearts), **p < 0.01, ***p < 0.001. (i–k) The localization and expression of FoxO3 in myocardium are detected by immunofluorescence staining. (i) Representative images for each condition are shown. (j) The quantification of FoxO3 fluorescence intensity is performed as described in Methods section. Results are presented as mean ± *SEM* (*n* = 21–25 sections from five hearts), ***p < 0.001. (k) Quantification of FoxO3^+^ cTnT^+^ cells in heart for each condition. Results are presented as mean ± *SEM* (*n* = 21–25 sections from 5 hearts), **p < 0.01

We next measured the in vivo effects of PQ exposure on FoxO3 activation in heart tissue (Figure 3a). Consistent with in vitro results shown in Figure S1A, PQ exposure significantly inhibited FoxO3 mRNA expression in heart tissue (Figure 3b). Using Western blotting analysis, we confirmed that the ratio of FoxO3 to β‐actin was greatly suppressed by PQ exposure compared with PBS treatment alone (Figure 3c,d), indicating that PQ exposure inhibits FoxO3 activation in vivo. Moreover, the absolute levels of FoxO3 phosphorylation were also reduced by PQ, although the ratio of phosphorylated FoxO3 to pan‐FoxO3 was elevated. (Figure 3c,e,f). Although no change for total Akt expression, the phosphorylation of Akt was significantly inhibited in PQ‐exposed heart compared with PBS treated alone (Figure 3c,g,h). These data suggest that PQ‐induced oxidative stress inhibits activation of the Akt/FoxO3 pathway in heart.

Given that phosphorylation of FoxO3 leads to nuclear exclusion, retention and degradation in cytoplasm, thereby deactivating the FoxO3 pathway (Brunet et al., 1999), the in vivo expression and localization of FoxO3 in cardiomyocytes were measured by immunofluorescence staining. As shown in Figure 3i, FoxO3 is mainly localized in the nuclei of cardiomyocytes, but the expression of FoxO3 was dramatically suppressed by PQ. Moreover, both the MFI of FoxO3 and the percentage of FoxO3^+^ cTnT^+^ cells were significantly reduced by PQ exposure (Figure 3j,k). These findings further confirm that PQ exposure suppresses FoxO3 activation in heart tissue.

### 2.5 Cardiac‐specific knockout of FoxO3 exacerbates PQ‐induced ventricular remodeling

**Figure 4:**
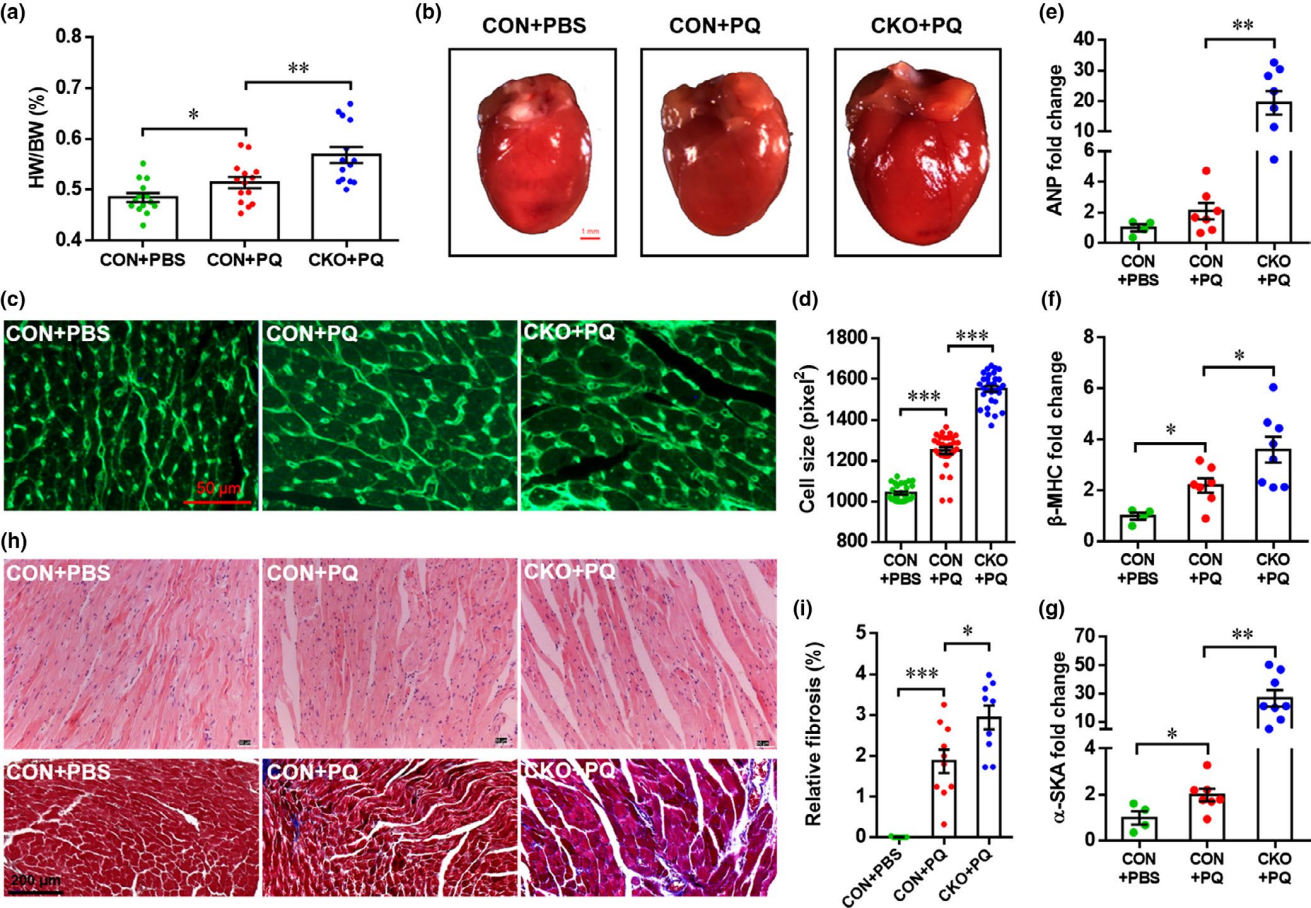
Cardiac‐specific knockout of Forkhead box O3 (FoxO3) exacerbates paraquat (PQ)‐induced cardiac hypertrophy and fibrosis. (a) The ratio of heart weight to body weight is analyzed. Results are presented as mean ± *SEM* (*n* = 14 heart per group), *p < 0.05, **p < 0.01. (b) Representative images of heart indicate FoxO3 knockout enhance PQ‐induced cardiac hypertrophy. (c and d) Cell size of cardiomyocytes is analyzed by WGA staining. (c) Representative images for each condition are shown. (d) Relative cell size of cardiomyocytes is quantified as described in Methods section. Results are presented as mean ± *SEM* (*n* = 30 sections from five hearts per group), ***p < 0.001. (e) Representative images of heart section with H&E staining (up pane) and Masson's trichrome staining (down panel). (f) Relative cardiac fibrosis is quantified as described in Methods section. Results are presented as mean ± *SEM* (*n* = 7–10 sections from five hearts per group), ***p < 0.001. (g–i) The mRNA expression of cardiac hypertrophy markers including atrial natriuretic peptide (ANP) (g), beta myosin heavy chain (β‐MHC) (h), and alpha‐skeletal actin (α‐SKA) (i) is analyzed by qRT–PCR. Results are presented as mean ± *SEM* (*n* = 4 hearts for CON + PBS, 7 hearts for CON + PQ, and 8 hearts for CKO + PQ), *p < 0.05, **p < 0.01

It has been demonstrated that oxidative stress plays an important role in cardiac hypertrophy and remodeling (Jin et al., 2017; Seddon, Looi, & Shah, 2007). To elucidate the potential role of FoxO3 in PQ‐induced cardiac damage, we generated cardiomyocyte‐specific *FoxO3* knockout (CKO) mice by crossing *FoxO3^f/f^* with *Myh6‐Cre* mice (Figure S3A). Both qRT–PCR (Figure S3B) and Western blotting (Figure S3C) using ventricular tissues confirmed the knockout of FoxO3. It was demonstrated that PQ exposure significantly increases the heart weight/body weight (HW/BW) ratio and heart size in CON mice. However, both the HW/BW ratio and heart size were further elevated in CKO mice compared with CON mice exposed to PQ (Figure 4a,b), suggesting that FoxO3 is necessary to protect against the cardiac hypertrophy induced by oxidative stress. Consistently, WGA staining demonstrated that PQ exposure increased the size of the cardiomyocytes in CON mice, and this was further increased in CKO mice (Figure 4c,d). More notably, the expression of hypertrophy markers, including atrial natriuretic peptide (*ANP*), beta myosin heavy chain (*β‐MHC*), and alpha‐skeletal actin (*α‐SKA*), was significantly increased by FoxO3 knockout in hearts treated with PQ (Figure 4e–g), further confirming the cardioprotective effects of FoxO3 in cardiac hypertrophy. Morphological analysis demonstrated that the PQ‐induced loss of adherence between cardiomyocytes was greatly exacerbated in CKO hearts compared with CON heats (Figure 4h, up panel). Indeed, PQ exposure can suppress the expression of N‐cadherin, one of proteins of adherence junctions, in cardiomyocyte cell lines (Figure S1H,I). In addition, PQ exposure induced cardiac fibrosis in CON hearts, which was further exacerbated by FoxO3 deletion specifically in cardiomyocytes (Figure 4h,i). Notably, FoxO3 knockout has no significant effects on the expression of hypertrophy markers in heart without PQ‐induced oxidative stress (Figure S4).

To evaluate the effects of FoxO3 on cardiac function under oxidative stress, cardiac function was evaluated by echocardiography after PQ administration. As shown in Figure S5A, the left ventricular (LV) systolic function was suppressed by PQ administration in CON mice, but the PQ‐induced decrease in LV systolic function was further enhanced. The LV ejection fraction (LVEF) was significantly decreased in CKO compared with CON mice after PQ administration (Figure S5B). Moreover, FoxO3 deficiency exacerbated PQ‐induced decline of LV fractional shortening (LVFS) (Figure S5C). Consistently, both the LV end‐diastolic dimension (LVEDd) and the LV end‐systolic dimension (LVESd) were significantly increased by PQ administration in CON mice, and these two values were further elevated in CKO mice (Figure S5D,E). In addition, cardiomyocyte‐specific knockout of FoxO3 significantly decreased the ventricular wall thickness in mice administered PQ as indicated by lower left ventricular posterior wall thickness of diastasis (LVPWTd), left ventricular posterior wall thickness of systole (LVPWTs), interventricular septal end‐diastolic thickness (IVSd), and interventricular septal end‐systolic thickness (IVSs) (Figure S5F–I). These findings suggest that cardiomyocyte‐specific knockout of FoxO3 exacerbates the cardiac dysfunction induced by PQ.

### 2.6 FoxO3 deletion exacerbates oxidative injury in hearts administered by PQ

**Figure 5:**
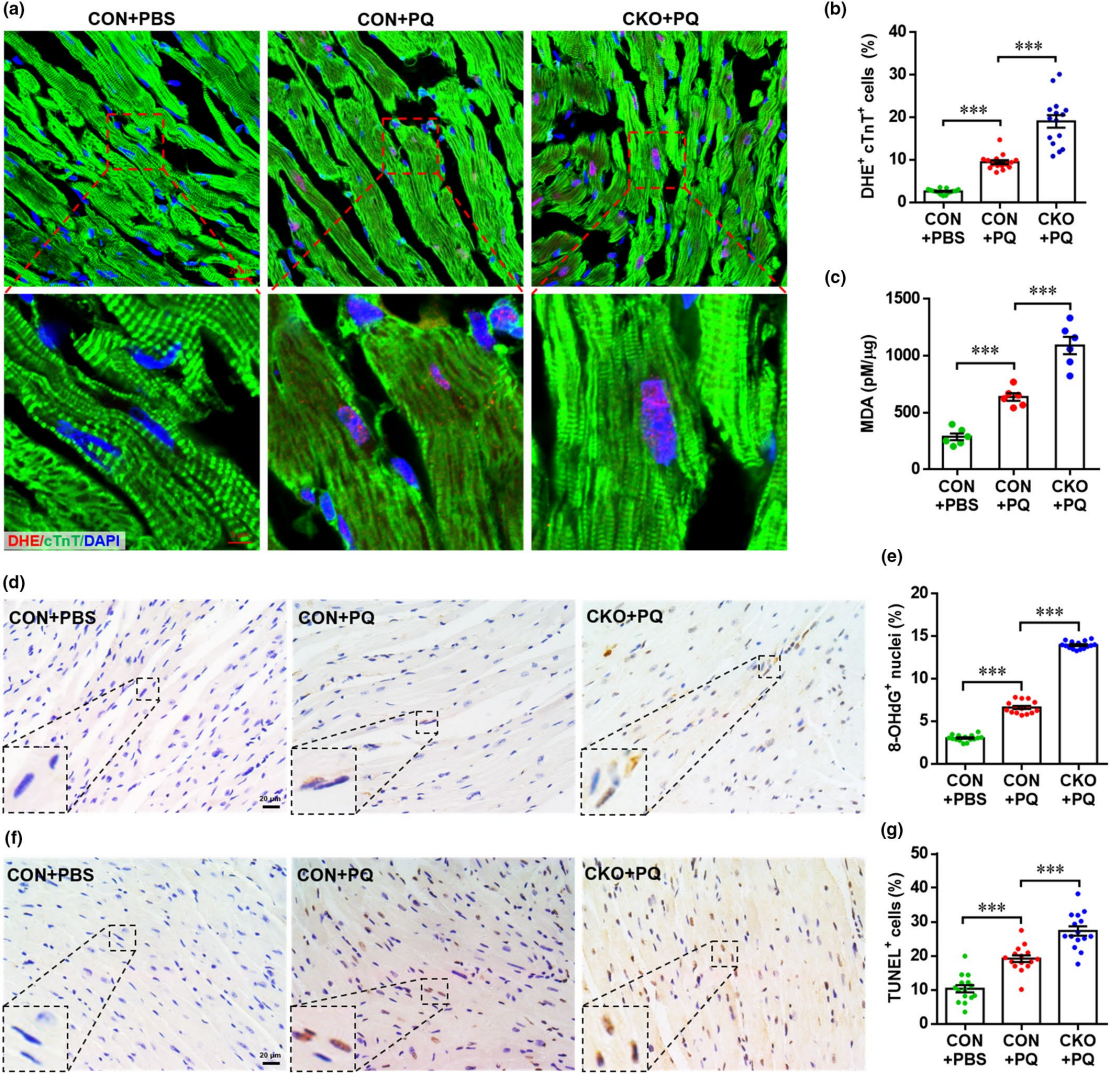
Cardiac‐specific knockout of Forkhead box O3 (FoxO3) exacerbates paraquat (PQ)‐induced oxidative damage in heart. (a) Representative images of dihydroethidium (DHE) staining showing that PQ‐induced reactive oxygen species (ROS) generation in cardiomyocytes are further enhanced by FoxO3 deletion. (b) Quantification of DHE^+^ cTnT^+^ cells in hearts for each condition. Values are presented as mean ± *SEM* (*n* = 15 sections from five hearts for each group), ***p < 0.001. (c) Quantification of malondialdehyde concentration in hearts from each group. Values are presented as mean ± *SEM* (*n* = 6 hearts for each group), ***p < 0.01. (d) Representative images of 8‐OHdG staining in hearts showing enhanced DNA damage in FoxO3 CKO mice compared with CON mice under PQ‐exposed conditions. (e) Quantification of 8‐OHdG^+^ cells in hearts for each condition. Values are presented as mean ± *SEM* (*n* = 15 sections from five hearts for each group), ***p < 0.001. (f) Representative images of TUNEL staining in hearts showing enhanced apoptosis in FoxO3 CKO mice compared with CON mice under PQ‐exposed conditions. (g) Quantification of TUNEL^+^ cells in hearts for each condition. Values are presented as mean ± *SEM* (*n* = 15 sections from five hearts for each group), ***p < 0.001

To examine oxidative stress in vivo, dihydroethidium (DHE) was used to evaluate ROS production in the hearts from CON and CKO mice, with or without PQ treatment. Consistent with the in vitro results (Figure 2b–d), the numbers of DHE^+^ cTnT^+^ cells were significantly increased by PQ exposure in CON hearts, but FoxO3 deficiency greatly elevated the number of DHE^+^ cTnT^+^ cells, indicating that FoxO3 deletion enhances ROS production in hearts exposed to PQ (Figure 5a,b). In addition, PQ administration markedly increased the level of oxidative stress in hearts from CON mice as determined by an elevated malondialdehyde (MDA) concentration, which was further elevated by FoxO3 deficiency (Figure 5c).

Given the evidence that oxidative DNA damage and apoptosis are commonly observed as oxidative stress‐dependent changes in the heart, and play important roles in oxidative stress‐induced cardiomyopathy (Nickel et al., 2015), oxidative DNA damage and apoptosis in myocardium were further examined. As shown in Figure 5d,e, staining for 8‐hydroxydeoxyguanosine (8‐OHdG), a biomarker for the effects of endogenous oxidative DNA damage, demonstrated the increased numbers of 8‐OHdG^+^ cells in hearts exposed to PQ, compared to resting hearts from CON mice. As expected, the percentage of 8‐OHdG^+^ cells in PQ‐stimulated hearts was further increased in the FoxO3 conditional knockout. In addition, PQ administration significantly increased apoptosis in myocardium as evidenced by the increased number of TUNEL^+^ cells, which was further increased by FoxO3 deficiency (Figure 5f,g). Taken together, these results suggest that the increased oxidative stress and myocardial damage induced by PQ are significantly aggravated by FoxO3 knockout specifically in cardiomyocytes.

### 2.7 Effects of FoxO3 on activation of the apoptotic and antioxidative pathways in hearts exposed to PQ

To further explore the mechanism by which FoxO3 protects heart from oxidative stress in vivo, the mRNA expression of antioxidant enzymes was examined by qRT–PCR. As shown in Figure S6A,B, the mRNA expression of *Cat* and *Sod2* was significantly increased in hearts from CON mice responding to PQ stimulation. However, the PQ‐induced increase in the mRNA expression of *Cat* and *Sod2* was strongly inhibited in the FoxO3 knockout, indicating that FoxO3 is an upstream positive regulator of these two antioxidant enzymes. These results were further confirmed at the protein level by Western blot analysis, which demonstrated that the expression of CAT and SOD2 in PQ‐stimulated hearts was remarkably suppressed by FoxO3 knockout (Figure S6C–E). We next examined the expression of apoptosis‐related proteins, including Bax, Bcl2, and p53 in vivo. Bax and p53 were significantly upregulated, but the expression of Bcl2 was downregulated in hearts from FoxO3 knockout mice exposed to PQ, compared with PQ‐treated CON mice. Moreover, the Bax/Bcl2 ratio was increased in hearts from CKO mice compared with PQ‐treated CON mice (Figure S6C,F–I). These findings further confirm that FoxO3 protects against PQ‐induced oxidative injury in hearts at least in part through anti‐apoptotic pathways. Consistent with the in vitro results, the expression of p16^INK4a^ was also greatly elevated in hearts with FoxO3 deletion, confirming that FoxO3 may play an important role in protection against oxidative stress‐induced aging in the heart. However, no significant difference in the expression of p21 and p27 was detected between CON and CKO mice treated with PQ (Figure S6C,J–L).

### 2.8 CAT and SOD2 contribute to FoxO3‐mediated cardiac protection from oxidative stress

**Figure 6:**
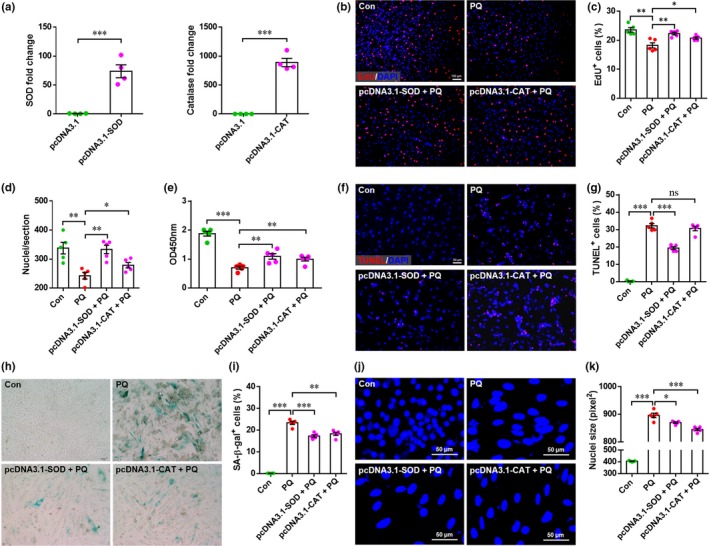
Both catalase (CAT) and superoxide dismutase 2 (SOD2) attenuate PQ‐induced senescent phenotype in Forkhead box O3 (FoxO3)‐silenced H9c2 cells. (a) shFoxO3‐H9c2 cells were transfected with pcDNA3.1‐SOD or pcDNA3.1‐CAT plasmid for 48 hr, respectively. The overexpression of target genes was analyzed by qRT–PCR analysis. The pcDNA3.1 empty plasmid was used as control. Values are presented as mean ± *SEM* (*n* = 4), ***p < 0.001. (b–d) The shFoxO3‐H9c2 cells were transfected with or without overexpression plasmids for 24 hr prior to treatment with PQ (400 μM) for another 4 hr, followed by proliferation analysis by 5‐ethynyl‐2′‐deoxyuridine (EdU) labeling. Representative images (b), quantification of EdU^+^ cells (c), and total cells (d) revealed that both SOD and CAT attenuate PQ‐induced inhibition of cell proliferation. Values are presented as mean ± *SEM* (*n* = 5), *p < 0.05, **p < 0.01. (e) The shFoxO3‐H9c2 cells were transfected with or without overexpression plasmids for 24 hr prior to treatment with PQ (400 μM) for another 24 hr, followed by cell growth activity analysis. Values are presented as mean ± *SEM* (*n* = 5), **p < 0.01, ***p < 0.001. (f and g) The shFoxO3‐H9c2 cells were treated with as mentioned above, followed by TUNEL labeling. Representative images (f) and quantification of TUNEL^+^ cells revealed that SOD, but not CAT, inhibits PQ‐induced apoptosis. Values are presented as mean ± *SEM* (*n* = 5), ***p < 0.01. (h–k) The shFoxO3‐H9c2 cells were transfected with or without overexpression plasmids for 24 hr prior to treatment with PQ (400 μM) for 3 days (4 hr/day), followed by senescence‐associated β‐galactosidase (SA‐β‐Gal) staining and nucleus staining, respectively. The representative images of SA‐β‐Gal^+^ cells (h) and total nuclei (j), and the quantification of SA‐β‐Gal^+^ cells (i) and nuclei size (k) are shown. Values are presented as mean ± *SEM* (*n* = 5), *p < 0.05, **p < 0.01, ***p < 0.001

Subsequently, we examined whether overexpression of *Cat* and *Sod2* could abolish the oxidative stress‐related phenotype of H9c2 cells with FoxO3 knockdown. As shown in Figure 6a, overexpression of *Cat* and *Sod2* was confirmed by qRT–PCR. 5‐Ethynyl‐2′‐deoxyuridine labeling results demonstrated that both the percentage of EdU^+^ cells and the total cell number were significantly elevated by forced expression of Sod2 or Cat compared with PQ treatment alone (Figure 6b–d), indicating the important role of antioxidant enzymes in the proliferation of H9c2 cells with FoxO3 knockdown. Consistently, the cell viability was also significantly increased by overexpression of *Cat* or *Sod2* in FoxO3‐deficient cells exposed to PQ (Figure 6e). The overexpression of *Sod2* significantly blocked PQ‐induced apoptosis in FoxO3‐deficient cells (Figure 6f,g). Moreover, both the percentage of SA‐β‐Gal^+^ cells and the nuclei sizes were significantly decreased by overexpression of *Cat* and *Sod2*, respectively (Figure 6h–k). Taken together, these findings further confirm that supplementation with antioxidant enzymes, including CAT and SOD2, blocks PQ‐induced oxidative stress‐related phenotypes in cardiomyocyte cell lines with FoxO3 deficiency.

To explore whether and how FoxO3 regulates the activation of CAT and SOD2 in mouse hearts, we analyzed the promoter sequences (−2,000 bp to −1 bp, upstream of TSS) of the mouse *Cat* (Gene ID: 12359) and *Sod2* (Gene ID: 20656) genes to determine potential FoxO3 binding sites. Bioinformatic analysis using JASPAR 2018 online software (http://jaspar.genereg.net/) (Khan et al., 2018) identified 21 and 23 potential FoxO3 binding sites for the *Cat* and *Sod2* promoters, respectively (Tables S1 and S2). The predicted binding site with the highest score for each promoter was further analyzed by chromatin immunoprecipitation (ChIP)‐qPCR, and an in vivo interaction between FoxO3 and the *Cat* and *Sod2* promoters in mouse heart was demonstrated (Figure S7), indicating that FoxO3 may transcriptionally activate *Cat* and *Sod2* in mouse hearts. This idea is supported by our previous reports demonstrating that FoxO3 significantly activates the transcription of the rat *Cat* and *Sod2* genes in luciferase reporter gene assays (Qi et al., 2015).

**Figure 7:**
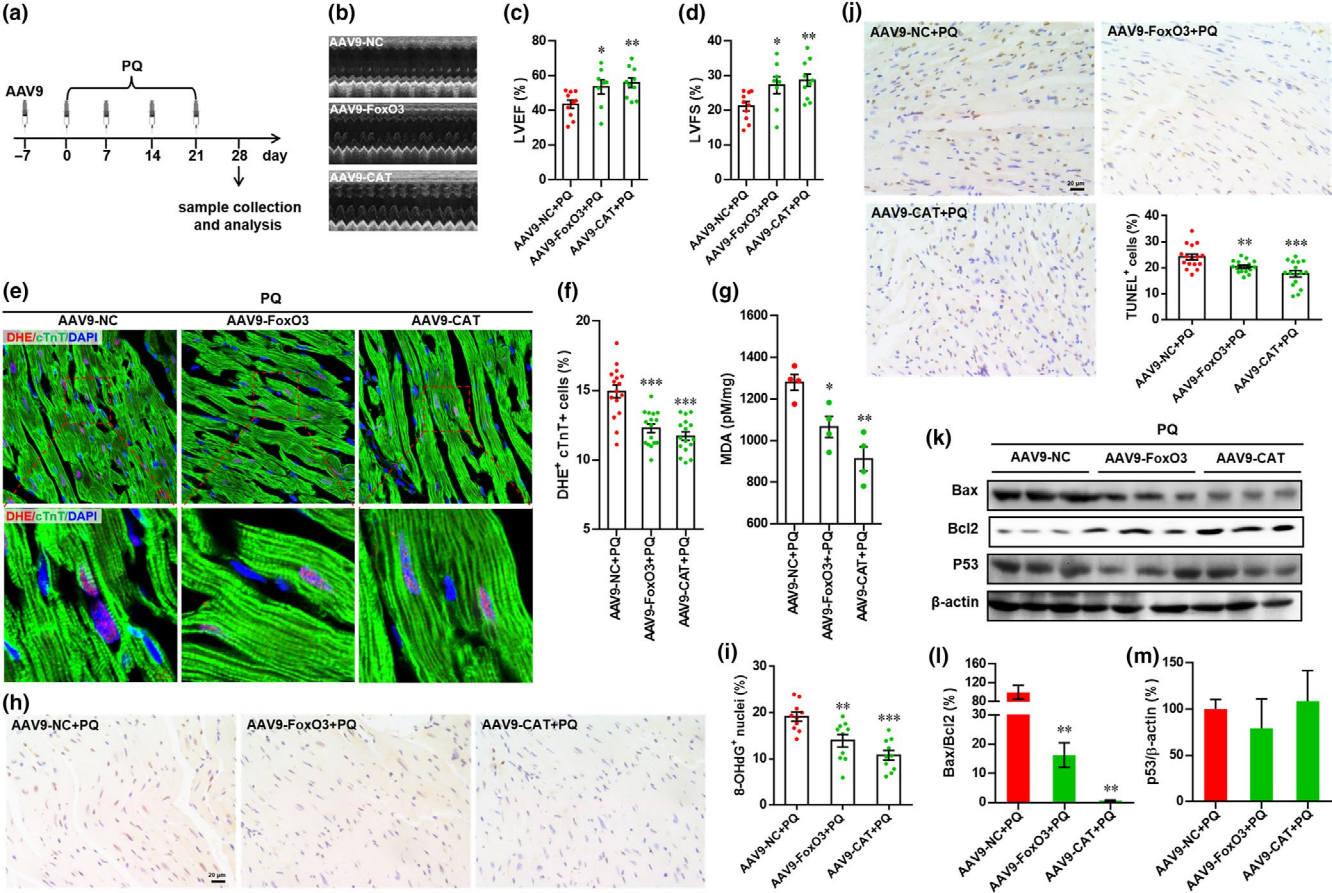
Overexpression of Forkhead box O3 (FoxO3) and catalase (CAT) attenuates PQ‐induced heart injury. (a) Schematic of AAV9 virus injection experiment designed to overexpress FoxO3 and CAT in hearts exposing to PQ. (b) Representative images of M‐model echocardiography. (c and d) The left ventricular ejection fraction (LVEF) (c) and left ventricular fractional shortening (LVFS) (d) are calculated by M‐mode echocardiography in each group. Values are presented as mean ± *SEM* (*n* = 8–10 hearts per group), *p < 0.05, **p < 0.01 versus control. (e) Representative images of dihydroethidium (DHE) staining. (f) Quantification of DHE^+^ cTnT^+^ cells in hearts for each condition. Values are presented as mean ± *SEM* (*n* = 16 sections from 8 hearts for each group), ***p < 0.001 versus control. (g) Quantification of malondialdehyde concentration in hearts from each group. Values are presented as mean ± *SEM* (*n* = 4 hearts for each group), *p < 0.05, **p < 0.01 versus control. (h) Representative images of 8‐OHdG staining in hearts. (i) Quantification of 8‐OHdG^+^ cells in hearts for each condition. Values are presented as mean ± *SEM* (*n* = 10 sections from 5 hearts for each group), **p < 0.01, ***p < 0.001 versus control. (j) Representative images of TUNEL staining and quantification of TUNEL^+^ cells in hearts for each condition. Values are presented as mean ± *SEM* (*n* = 16 sections from 8 hearts for each group), **p < 0.01, ***p < 0.001 versus control. (k) Representative images of Western blotting for Bax, Bcl2, and p53. (l and m) The relative expression levels of target proteins are quantified as the percentage of control. Results are presented as mean ± *SEM* (*n* = 3 hearts), **p < 0.01 versus control

Compared with *Sod2* promoter, *Cat* promoter showed higher enrichment in FoxO3 proteins (Figure S7C–E). Thus, *Cat* was further examined in vivo. As shown in Figure 7a, both CAT and FoxO3 were overexpressed by AAV9 virus system in hearts, which was followed by PQ exposure. Overexpression of target genes was confirmed by qRT–PCR (Figure S8A). As expected, cardiac function was significantly increased by FoxO3 and CAT overexpression in PQ‐treated mice (Figure 7b–d). The PQ‐induced ventricular remodeling was suppressed by overexpression of FoxO3 and CAT, respectively, which was demonstrated by decreased HW/BW ratio, cardiomyocyte size, and fibrosis (Figure S8B–G). Moreover, forced expression of FoxO3 and CAT suppressed the PQ‐induced oxidative stress in hearts, which was demonstrated by decreases in DHE^+^ cTnT^+^ cell number (Figure 7e,f), MDA production (Figure 7g), and 8‐OHdG^+^ cell number (Figure 7h,i). In addition, cell apoptosis evaluation demonstrated that FoxO3 and CAT forced expression in hearts significantly suppressed PQ‐induced cell apoptosis, which was demonstrated by the decreases in TUNEL^+^ cell numbers (Figure 7j) and Bax/Bcl2 ratio (Figure 7k–m).

## 3 DISCUSSION

Aging is a major risk factor for cardiovascular disease, which is the leading cause of mortality worldwide. Cardiac aging is a complex pathophysiological process that exhibits unique histological and biochemical characteristics including cardiac remodeling and dysfunction (Boengler, Schulz, & Heusch, 2009; Hua et al., 2015; Lakatta & Levy, 2003). The precise mechanisms contributing to cardiac aging are far from clear, but several postulates have been proposed including oxidative stress (Yang et al., 2006). Paraquat is a quaternary nitrogen herbicide and strong ROS generator. Data from several laboratories suggest that PQ‐induced oxidative stress suppresses myocardial survival, impairs myocardial contractive function, and promotes heart failure (Ge et al., 2010; Vinciguerra et al., 2012; Wang et al., 2017). In the present study, we evaluated the effect of FoxO3 on PQ‐induced cardiac dysfunction and explored the potential mechanisms. Both in vitro and in vivo results from this study indicate that FoxO3 deficiency aggravates PQ‐induced heart injury by suppressing oxidative stress via the upregulation of antioxidant enzymes including Cat and Sod2. We also demonstrated that FoxO3 deficiency promotes the PQ‐induced phenotype of cardiac aging as evidenced by ventricular remodeling, cardiac dysfunction, apoptosis, and senescence.

Paraquat is a ROS generator that causes oxidative stress, which has been demonstrated to induce cellular senescence in replication‐competent cell types (Campisi, 2013). Indeed, the contribution of PQ treatment to senescence induction has been demonstrated in cultured human fibroblasts (Jung et al., 2009; Wiley et al., 2016) and human astrocytes (Chinta et al., 2018). Moreover, in vivo data have demonstrated that systemic PQ exposure induces astrocytic senescence and SASP in the brain, thereby promoting neuropathology (Chinta et al., 2018). These previous studies suggest that PQ is suitable for inducing senescence in cultured cells. In the present study, our results demonstrate that PQ exposure induces senescence‐associated phenotypes in cultured cardiomyocyte cell lines, including increasing ROS generation, apoptosis, SA‐β‐Gal activity, and p16^INK4a^ expression as well as decreasing cell growth and proliferation. Importantly, administration of PQ to mice induced representative cardiac remodeling as evidenced by increases in the HW/BW ratio, heart size, cardiomyocyte size, expression of hypertrophy‐related marker genes, and fibrosis in myocardium. Moreover, PQ administration also induced cardiac dysfunction in mice, including reducing cardiac contractile function, oxidative myocardium damage, and cardiomyocyte apoptosis. These findings are consistent with previously reported phenotypes associated with cardiac aging (Boengler et al., 2009; Hua et al., 2015). Taken together, these findings indicate that systemic PQ administration induces cardiac aging in addition to cellular senescence in the brain as previously reported (Chinta et al., 2018). To our knowledge, this is the first study to suggest aging‐associated cardiac anomalies as a response to PQ administration.

Forkhead box O3 is a longevity factor that has been implicated in human lifespan extension (Flachsbart et al., 2017; Martins et al., 2016). Given that FoxO3 is evolutionally conserved, it is possible that FoxO3 prevents the aging process of organs and cells in different organisms. Indeed, cardiac‐specific overexpression of dFoxO prevents the age‐dependent decline in heart function in *Drosophila* (Wessells et al., 2004). Moreover, an age‐mediated decrease in FoxO3 activity was observed in aged rats (Choi et al., 2012). These studies suggest that the longevity factor FoxO3 may prevent the aging process of organs and cells even in mammals. This idea is further supported by data from the present study. For cardiomyocyte cell lines, our results demonstrated that FoxO3 knockdown significantly enhances PQ‐induced senescence‐associated phenotypes as evidenced not only by increased SA‐β‐Gal activity, p16^INK4a^ levels, apoptosis levels, and ROS generation, but also by decreased levels of cell growth and proliferation (Figures 1, 2, S1 and S2). Decreased FoxO3 activation was also detected in senescent H9c2 cells induced by PQ exposure (Figure S1). It has been reported that PQ can induce myocardial mitochondrial injury (Wang et al., 2017). Consistently, PQ exposure indeed decreased ΔΨm in cardiomyocyte cell lines, evidenced by the JC‐1 staining. Notably, FoxO3 deficiency further reduced the ΔΨm in PQ‐exposed cells, indicating that FoxO3 is crucial to attenuate PQ‐induced apoptosis in cardiomyocyte cell lines (Figure 2h). Furthermore, PQ administration to mice greatly suppressed the activity of FoxO3 in the heart (Figure 3), indicating that FoxO3 deficiency may enhance the cardiac aging phenotypes induced by PQ. As expected, there was increased cardiac remodeling, dysfunction, oxidative damage, apoptosis, and senescent hallmark (p16^INK4a^) expression in the cardiomyocyte‐specific FoxO3 knockout hearts exposed to PQ (Figures 4, 5, S5 and S6). Thus, our in vitro and in vivo findings reveal that FoxO3 plays an important role in protecting against the cardiac aging induced by PQ administration. This hypothesis was further supported, at least in part, by a previous study showing that FoxO3 activity was significantly decreased in aging kidney isolated from 21‐month‐old mice compared with young mice (Choi et al., 2012).

Previous studies from our group and others have documented that FoxO3 plays a protective role in resistance to oxidative stress in multiple cell types through the upregulation of antioxidant enzymes including CAT and SOD2, and other survival pathways (Lim et al., 2017; Qi et al., 2015). Overexpression of catalase has been demonstrated to extend lifespan and retard aging‐induced cardiac dysfunction in mice through an antioxidant mechanism (Schriner et al., 2005), suggesting that a reduction in oxidative stress plays an important role in suppressing the aging of organs and organisms. These previous studies are consistent with our findings demonstrating that FoxO3 deficiency exacerbates PQ‐induced phenotypes in vitro and in vivo, which were accompanied by decreased activation of CAT and SOD2 and increased oxidative stress (Figures 2, 5, S2 and S6). More importantly, overexpression of *Cat* or *Sod2* significantly blocked senescence‐associated phenotypes including inhibition of proliferation and growth and the induction of SA‐β‐Gal activity and apoptosis in PQ‐treated H9c2 cells with FoxO3 knockdown (Figure 6). Therefore, it is reasonable to conclude that CAT and SOD2 are at least in part responsible for FoxO3‐mediated protection against the cardiac aging induced by PQ. In the present study, we further demonstrate that FoxO3 can directly bind to the promoter regions of the mouse *Cat* and *Sod2* genes in heart tissue as evidenced by ChIP analysis (Figure S7). Importantly, both FoxO3 and CAT overexpression in vivo greatly suppressed PQ‐induced heart injury and aging‐associated phenotypes (Figures 7 and S8).

In conclusion, our data show that systemic administration of PQ induced phenotype‐ associated cardiac aging, which were further exacerbated by FoxO3 deficiency. We demonstrate that the protective role of FoxO3 in PQ‐induced cardiac aging is attributed to upregulation of antioxidative enzymes, including *Cat* and *Sod2*, by directly binding to their promoters, thereby suppressing oxidative stress and protecting against cardiac aging‐associated phenotypes, including cardiac remodeling, apoptosis, oxidative damage, and dysfunction. This finding provides novel links among environmental exposure, FoxO3 deficiency, and cardiac aging. Further studies are necessary to explore the potential cardiac protective role in natural aging mouse models. Our results suggest that the longevity factor FoxO3 might be a potential therapeutic target for cardiac aging.

## 4 EXPERIMENTAL PROCEDURES

All animal protocols and procedures were approved by the Laboratory Animal Committee of Jinan University. For all detailed Experimental Procedures, see Appendix S1.

## CONFLICT OF INTEREST

None declared.

## AUTHOR CONTRIBUTIONS

XF Qi, ZS Chang, JB Xia, and HY Wu designed the study, performed most experiments, and analyzed the data. WT Peng, FQ Jiang, J Li, and CQ Liang contributed animal and cellular experiments as well as data analysis. H Zhao, KS Park, GH Song, SK Kim, and R Huang provided valuable comments and revised and edited the manuscript. XF Qi, DQ Cai, and L Zheng conceived of and supervised the study. XF Qi and ZS Chang wrote the manuscript.
