# Supplementary material for: Forkhead box O3 protects the heart against paraquat‐induced aging‐associated phenotypes by upregulating the expression of antioxidant enzymes
Source: Aging Cell. 2019 Jul 1;18(5):e12990. doi: 10.1111/acel.12990 (PMC6718552; doi:10.1111/acel.12990)
Supplement: Supplementary file 1 [file ACEL-18-e12990-s001.docx]

**Supporting Information**

**Supporting Experimental Procedures**

**Cell culture**

H9c2 cells (rat cardiomyocyte cell line) were purchased from FuDan IBS Cell Center (FDCC, Shanghai, China) and were cultured in high glucose DMEM (HyClone, USA) with 10% fetal bovine serum (Gibco, USA), l µg/mL streptomycin (Gibco, USA), and 1 U/mL penicillin streptomycin (Gibco, USA), at 37℃ in a 5 % CO_2_ incubator. Culture medium was replaced every 2 to 3 days and cells were passaged when they reached 80% confluence.

**Establishment of stable FoxO3 knockdown cells**

The shRNA expression lenti-viral vector pLOX-U6-Puro was constructed using standardized molecular cloning technology, based on the pLOX-U6-E/P vector (Qi *et al.* 2015). In brief, the EF1α-Puro cassette flanked by *BamHI* and *KpnI* restriction enzyme sites was generated by fusion PCR using the pLOX-U6-E/P template. At the same time, pLOX-U6-E/P vector was digested with the *BamHI* and *KpnI* to remove the EF1α-E/P cassette. The isolated vector backbone was then ligated with the amplified EF1α-Puro cassette to generate pLOX-U6-Puro vector. Stable knockdown of FoxO3 gene in H9c2 cells was achieved by lenti-viral based short-hairpin RNA delivery. FoxO3 specific shRNA or negative control were cloned into pLOX-U6-Puro vectors. Viral particles were packaged in HEK 293T cells and used to infect H9c2 cells as previously described (Qi *et al.* 2015). Infected H9c2 cells were selected by puromycin and expanded to form a stable sub-line. Knockdown efficiency was confirmed at both mRNA and protein levels. The shRNA sequences are as follows: FoxO3-shRNA: 5′-GGA ACT TCA CTG GTG CTA AGC-3′; negative shRNA (NC): 5′-ACT ACC GTT GTT ATA GGT G-3′. Stable cell line generated by FoxO3-shRNA and negative shRNA were named as shFoxO3 cells and shNC cells, respectively.

**Cell proliferation assay**

H9c2 cells were incubated with PQ (0-1 mM) for 24 hr, followed by cell viability analysis using the Enhanced Cell Counting Kit-8 (CCK-8, Beyotime Biotechnology, China) according to the manufacturer’s instructions, as previously described (Zhou *et al.* 2018). Moreover, H9c2 cells were incubated with or without PQ (400 µM) for 4 hr. DNA synthesis were then analyzed by 5-Ethynyl-2’-deoxyuridine (EdU) labelling, using Cell-Light™ EdU Apollo®567 In Vitro Imaging Kit (RiboBio, Guangzhou, China) according to the manufacturer’s instructions. The population of EdU positive cells was determined by counting at least 600 cells per dish and images were taken using a phase-contrast microscope (Olympus, Japan). The EdU positive cells were quantified as the percentage of total cells.

**Senescence-associated β-galactosidase (SA-β-Gal) staining**

H9c2 cells were incubated with PQ (400 µM) for 4 hr, cultured medium was then replaced by fresh medium. PQ treatment was performed once per day for total 3 days. Senescence-associated β-galactosidase (SA-β-Gal) activity was then detected using the Senescence β-Galactosidase Staining Kit (Beyotime Biotechnology, China) according to the manufacturer's instructions, as previously described (Qi *et al.* 2015). The population of SA-β-Gal positive cells was determined by counting at least 500 cells per dish and images were taken using a phase-contrast microscope (Olympus, Japan). The SA-β-Gal positive cells were quantified as the percentage of total cells. Image-Pro Plus version 6.0 software was used to quantify the size of each cell.

**Determination of intracellular ROS in H9c2 cells**

The intracellular ROS generation was evaluated by measuring the oxidation of CM-H_2_DCFDA (5-(and-6)-chloromethyl-2’,7’-dichlorofluorescein diacetate, Molecular Probes, Invitrogen, USA) with flow cytometry. In brief, H9c2 cells were incubated in DMEM with or without paraquat 400 µM (Sigma, USA) for 4 hr, followed by washing 3 times with phosphate-buffered saline (PBS), cells were then incubated 20 min in the dark with 10 µM CM-H_2_DCFDA probe. Cells were washed again with PBS and were monitored with a flow cytometer (BD Biosciences, USA) at an excitation wavelength of 488 nm and an emission wavelength of 525 nm. Intracellular ROS concentration was determined by the mean fluorescence intensity (MFI) of 10,000 cells, as well as by the percentage of DCF-positive cells.

**Measurement of mitochondrial membrane potential (ΔΨm)**

Mitochondrial membrane potential was detected by JC-1 (BD Biosciences, USA) staining as described previously (Qi *et al.* 2013), with some modification. Treated cells were collected, washed with PBS, and then incubated with 10 µM JC-1 in the dark for 20 min at 37℃, according to the manufacturer’s instructions. Stained cells were washed and resuspended with PBS, and then subjected to flow cytometry analysis on a flow cytometer (BD Biosciences, USA). The values of mitochondrial membrane potential from each sample were expressed as ratios of red fluorescence intensity to green fluorescence intensity.

**Apoptosis determination in H9c2 cells**

Phosphatidylserine on the cell surface was detected with Annexin V-FITC Apoptosis Detection Kit I (BD Biosciences, USA) according to the manufacturer’s instructions, as previously described (Qi *et al.* 2013). Briefly, paraquat-treated H9c2 cells were washed three times with PBS, suspended in 100 µl annexin-V binding buffer, and followed by incubation with 2.5 µl Annexin V-FITC and 2.5 µl PI for 10 min at room temperature in the dark. Cells were washed with PBS, suspended in binding buffer, and subjected to flow cytometry analysis (BD Biosciences, USA) with 488 nm excitation and 620 nm emission filters. Both early (Annexin V^+^/PI^-^) and later (Annexin V^+^/PI^+^) apoptotic cells were quantified and expressed as a percentage of total cells examined.
**Overexpression vector construction**

Total RNA was isolated using RNeasy Kit (Qiagen, Valencia, CA, USA) from H9c2 cells according to the manufacture’s instruction. The cDNA was transcribed from total RNA, using SuperScript III Reverse Transcriptase (Roche, USA). The coding sequences (CDS) of *Cat* (NM_012520.2) and *Sod2* (NM_017051.2) were amplified by KOD-Plus-Neo Kit (Toyobo, Japan) and cloned into pcDNA3.1 expression vector as mentioned previously (Xia *et al.* 2016). Primer sequences used for T vector cloning are as follows: Cat-F1: 5’-CCC TCT TGC CTC ACG TTC TG-3’ and Cat-R1: 5’-ATC TCC TCA GTG CAG GCT GA-3’; Sod2-F1: 5’-CCG TGT TCT GAG GAG AGC AG-3’ and Sod2-R1: 5’-TAC AAA ACA CCC ACC ACG GG-3’. Primer sequences containing restriction enzyme sites for sub-cloning are as follows: Cat-F-*NheI*: 5’-CTA *GCT AGC* ATG GCG GAC AGC CGG GAC CC-3’ and Cat-R-*XhoI*: 5’-CC*C TCG AG*T TAC AGG TTA GCT TTT CCC T-3’; Sod2-F-*EcoRI*: 5’-CG*G AAT TC*A TGT TGT GTC GGG CGG CGT G-3’ and Sod2-R-*XhoI*: 5’-CC*C TCG AG*T CAC TTC TTG CAA ACT ATG T-3’. Constructed overexpression vectors pcDNA3.1-Cat and pcDNA3.1-Sod2 were further confirmed by DNA sequencing. Overexpression vectors were transfected into shFoxO3 cells exposed to PQ to explore the involvement of target genes in FoxO3-mediated antioxidative functions.

**Animals**

All animal protocols and procedures were approved by the Laboratory Animal Committee of Jinan University. All experiments were performed on age-matched male mice. FoxO3-LoxP-targeted (*FoxO3^fl/fl^*) mice (C57BL/6 background) were created by Cyagen Biosciences (Suzhou, China). The exon 3 region of the FoxO3 gene was flanked by LoxP sites and deleted upon Cre-mediated recombination (Fig. S3). Mice were genotyped using specific PCR primers designed for the LoxP site: FoxO3fl-Forward: 5’-CAG CCG GTT CAT CAG AGT TTT ACC-3’; FoxO3fl-Reverse: 5’- CTA TCA ACG AGG TAG TGA TCT AAG AAC ATG C-3’. The wild-type (WT) allele was identified as a 339 bp PCR product and the mutant allele was determined by a 467 bp PCR product on a 1.5% agarose gel. The *Myh6-Cre* transgenic line was obtained from the Jackson Laboratory (stock number 011038). *Myh6-Cre* mice were genotyped using 9543 (5’-ATG ACA GAC AGA TCC CTC CTA TCT CC-3’) and 9544 (5’-CTC ATC ACT CGT TGC ATC ATC GAC-3’) primers and identified by a 300 bp PCR product. The *FoxO3^fl/fl^::Myh6-Cre* (CKO) mice were generated by crossing *FoxO3^fl/fl^* mice with *Myh6-Cre* mice. Age-matched *Myh6-Cre* mice were used as control (CON).

**PQ administration**

Mice at 4-5 months of age were randomized to receive an intraperitoneal (i.p.) injection of PQ at 15 mg/Kg body weight concentration (Sigma, USA). Mice were injected once per week, for total 4 times. Animals were subjected to echocardiography and other related analysis 7 days after the last PQ administration. The injection of equal volume of PBS was used as control.

**Echocardiography**

After treatment with PQ or PBS, the cardiac function was evaluated using the Vevo® 2100 ultrasound system (Visualsonics, Toronto, Canada) equipped with a high-frequency (30 MHz) linear array transducer, as described previously (Xia *et al.* 2017).

**Histology**

Following echocardiography analysis, mice were sacrificed and weighed to obtain total body weight (BW). The heart was harvested and weighed to obtain heart weight (HW). Cardiac hypertrophy was determined by the ratio of HW/BW. Heart tissues were fixed in 4% formaldehyde and dehydrated in a series of ethanol. The fixed heart was embedded in paraffin and sectioned with 5 μm thickness. Sections were subjected to hematoxylin and eosin stain (H&E) staining and Masson’s trichrome staining for morphological and fibrosis analysis, respectively. The fibrotic scar size was measured using Image-Pro Plus version 6.0 software and expressed as a percentage of ventricular area.

**Wheat germ agglutinin (WGA) staining and quantify cell size**

Sections were deparaffinized, rehydrated, rinsed three times in PBS and fixed in 4% paraformaldehyde for 15 minutes at 37°C. After washing three times in PBS, sections were then incubated for 10 minutes at room temperature with primary antibody against WGA conjugated to Alexa Fluor 488 (5 μg/mL, Invitrogen, USA). Sections were rinsed three time in PBS and mounted in Antifade Mounting Medium (Jackson ImmunoResearch Laboratories, USA). To quantify the cell size, five independent hearts (about 2,000 cells from 6 sections each heart) per group were captured with laser-scanning confocal microscope (LSM 700, Zeiss). Image-Pro Plus version 6.0 software was used to quantify the size of each cell.

**Immunofluorescence staining**

Heart tissues were embedded in Tissue-Tek optimal cutting temperature compound (OCT) (Sakura, USA) for frozen section. Frozen sections (5 µm) were permeabilized with 0.5% Triton X-100 in PBS, blocked with 5% goat serum (Jackson ImmunoResearch Laboratories, USA) for 1 hour at room temperature, and incubated with primary antibodies overnight at 4℃. The sections were subsequently washed with PBS and incubated with corresponding secondary antibodies conjugated to fluorescence for 1 hour at room temperature, followed by counterstaining with DAPI (Sigma, USA). Primary antibodies used are following: anti-FoxO3 (1:500 dilution, Cell Signaling Technology, USA) and anti-cTnT (1:100 dilution, Cell Signaling Technology, USA). Secondary antibodies used are following: Alexa Fluor 488 goat anti-rabbit IgG (1:200 dilution, Jackson ImmunoResearch Laboratories, USA) and Cy3-conjugated AffiniPure Goat anti-mouse IgG (1:100 dilution, Proteintech, USA). The slides were imaged with Zeiss LSM 700 laser confocal microscope (Carl Zeiss). Fluorescent signals were quantitated with Image-Pro Plus version 6.0 software.

**TUNEL Staining**

Hearts were fixed in 4% paraformaldehyde overnight at room temperature and embedded in paraffin as above described. Sections (5 μm) were stained by terminal deoxynucleotidyl transferase-mediated dUTP nick end labeling (TUNEL) and diaminobenzidine (DAB) staining kit (KeyGEN Biotech, Nanjing, China) to detect apoptotic cells according to the manufacturer’s instructions. Sections were counterstained with hematoxylin solution (Servicebio, Wuhan, China). The percentage of TUNEL-positive nuclei was counted in 15 sections from 5 hearts per group (3 sections per heart). Apoptosis index (percentage of TUNEL-positive nuclei) was calculated as TUNEL-positive nuclei/total nuclei × 100 (%).

**8-OHdG staining**

Paraffin-embedded tissue sections were dewaxed in xylene and dehydrated in an alcohol gradient. Endogenous peroxidase activity was quenched with 3% hydrogen peroxide and antigen retrieval was achieved by heating slides covered with citrate buffer (pH =6.0, Servicebio, Wuhan, China) at 95℃ for10 min. Slides were blocked with 5% BSA, and then incubated with primary antibody for 8-OHdG (Abcam, USA) overnight at 4°C. Sections were extensively washed in PBS and then incubated with HRP-conjugated secondary antibody (ServiceBio, Wuhan, China) for 1 h at room temperature. Cell nuclei were counterstained with hematoxylin solution, and immunoreactivity was visualized with DAB (Servicebio, Wuhan, China). Negative controls were obtained by incubating sections with PBS instead of primary antibody. Brown particles present in the nuclei was considered positive signals and quantified by Image-Pro Plus version 6.0 software. The percentage of 8-OHdG-positive nuclei was counted in 15 sections from 5 hearts per group (3 sections per heart).

**Isolation of total mRNA and quantitative real-time PCR**

Total RNA was isolated using RNeasy Kit (Qiagen, Valencia, CA, USA) from H9c2 cells and heart tissue according to the protocol of the manufacturer, respectively. Reverse transcription to cDNA was performed with 30 ng of total RNA, random primers, and SuperScript III Reverse Transcriptase (Roche, USA). Real-time PCR was performed using a Light Cycler 480 SYBR Green I Master (Roche, USA) and the MiniOpticon Real-Time PCR System (Bio-Rad, CA, USA). The sequences of the PCR primers were as follows: FoxO3-F: 5’-CGT TCC TGA AGG GAA GGA G-3’ and FoxO3-R: 5’-GCT TGG GCT CTT GCT CTC T-3’; ANP-F: 5’-GGG CTT CTT CCT CGT CTT GG-3’ and ANP-R: 5’-GGT CTA GCA GGT TCT TGA AAT CC-3’; β-MHC-F: 5’- ACT GTC AAC ACT AAG AGG GTC A-3’ and β-MHC-R: 5’-TTG GAT GAT TTG ATC TTC CAG GG-3’; α-SKA-F: 5’-CCC AAA GCT AAC CGG GAG AAG-3’ and α-SKA-R: 5’-CCA GAA TCC AAC ACG ATG CC-3’; CAT-F: 5’-AGC GAC CAG ATG AAG CAG TG-3’ and CAT-R: 5’-TCC GCT CTC TGT CAA AGT GTG-3’; SOD2-F: 5’-CAG ACC TGC CTT ACG ACT ATG G-3’ and SOD2-R: 5’-CTC GGT GGC GTT GAG ATT GTT-3’; GAPDH-F: 5’-TGT GTC CGT CGT GGA TCT GA-3’ and GAPDH-R: 5’-CCT GCT TCA CCA CCT TCT TGA-3’. After denaturation for 10 min at 95 °C, the reactions were subjected to 45 cycles of 95 °C for 30 s, 60 °C for 30 s, and 72 °C for 30 s. GAPDH was used as the internal standard control to normalize gene expression using the △△Ct method.

**Detection of ROS generation *in vivo***

To evaluate ROS generation in heart tissue, fresh and frozen left ventricular myocardium (5 µm sections) was incubated with dihydroethidium (DHE, 1 mmol/L, Molecular Probes, USA) for 1 hr at room temperature. Following 3 times washing by PBS, the sections were stained with DAPI (1 µg/mL, Sigma, USA) for 5 min at room temperature. Slides were rinsed three times with PBS and cover slipped. The numbers of DHE-positive nuclei and the total nuclei were counted in 15 sections from 5 hearts per group (3 sections per heart). *In vivo* ROS generation was quantified by the percentage of DHE-positive nuclei.

**MDA assessment in heart**

Malondialdehyde (MDA) is a terminal product of lipid peroxidation. For the MDA assay, proteins in heart were prepared according to the description of the Lipid Peroxidation MDA assay kit (Beyotime Biotechnology, Haimen, China). The MDA levels were detected by a microplate reader (Bio-tek Instruments, VT, USA) at 532 nm, according to the manufacturer’s instructions of Lipid Peroxidation MDA assay kit (Beyotime Biotechnology, Haimen, China).

**Western blotting**

H9c2 cells and heart tissues for SDS-PAGE were lysed in RIPA buffer (Beyotime Biotechnology, Haimen, China) containing protease (Sigma, USA) and phosphatase inhibitors (Thermo Fisher, USA). Protein concentration ware determined using the Bio-Rad Protein Assay (Bio-Rad Laboratories). Proteins (30 μg) were separated by SDA-PAGE, transferred onto PVDF membrane (Millipore), blocked in 5% nonfat milk/TBS-Tween 20, and incubated with primary antibodies (dilution in TBST) at a pre-determined optimal concentration overnight at 4℃. After rinsed with TBS containing 0.1% Triton X-100 (TBST), membrane was then incubated with corresponding second antibodies for 1 hour at room temperature. Bands were detected by chemiluminescence reagents (Thermo Fisher, USA). Primary antibodies used in this study are following: anti-FoxO3 (CST, 1:1000 dilution), anti-pFoxO3-Ser253 (Abcam, 1:500 dilution), anti-pFoxO3-Thr32 (Santa Cruz, 1:100 dilution) , anti-pAkt (CST, 1:1000 dilution), anti-Akt (CST, 1:1000 dilution), anti-SOD2 (Abcam, 1:1000 dilution), anti-CAT (CST, 1:1000 dilution), anti-p53 (Abcam, 1:1000 dilution), anti-p16^INK4a^ (Abcam, 1:1000 dilution) , anti-p21 (Abcam, 1:1000 dilution), anti-p27 (CST, 1:1000 dilution) , anti-Bax (CST, 1:1000 dilution), anti-Bcl2 (CST, 1:1000 dilution), anti-N-cadherin (CST, 1:1000 dilution) and anti-β-actin (Proteintech, 1:4000 dilution). Secondary antibodies used are following: goat-anti-mouse horseradish peroxidase (HRP)-conjugated antibody (CST, 1:4000); goat-anti-rabbit HRP-conjugated antibody (CST, 1:2000). Chemiluminescent signals were quantitated with Image-Pro Plus version 6.0 software.

**Chromatin immunoprecipitation (ChIP) assay**

The promoter sequences (-2,000 bp to -1 bp, upstream of TSS) of the mouse *Cat* (Gene ID: 12359) and *Sod2* (Gene ID: 20656) genes were analyzed by JASPAR 2018 online software (http://jaspar.genereg.net/) (Khan *et al.* 2018) to determine potential FoxO3 binding sites (Tables S1 and 2). The predicted binding site with highest score for each promoter was further analyzed by ChIP assay, to evaluate the *in vivo* binding of FoxO3 to its consensus sequence in mouse *Cat* and *Sod2* promoters. The assays were done in whole mouse hearts using the SimpleChIP® Plus Enzymatic Chromatin IP Kit (CST, #9004) as described previously (Mahmoud *et al.* 2013). FoxO3 antibody (Abcam, #ab12162) was used. Normal goat IgG (CST, #2729) was used as a control as previously described. The DNA isolated from input chromatin fragments and from the precipitated chromatin fragments by anti-FoxO3 antibody or control IgG was subjected to PCR using primers flanking the consensus FoxO3 binding sites on *Cat* and *Sod2* promoters. PCR products were determined on a 1.5% agarose gel. Relative binding ability of FoxO3 was expressed as the DNA signals relative to input. ChIP-PCR primers used in this study as follows: Cat-ChIP-F: 5’-AAA TAA GCT GCA AAG CCA CCA A-3’ and Cat-ChIP-R: 5’-CAT AGC TCC TTT GAG ACC AGA C-3’; Sod2-ChIP-F: 5’-GAG CCA TGG GAT ACG TGC TAA-3’ and Sod2-ChIP-R: 5’-GCT GAT GTC AAA GCG GTC TT-3’.

**FoxO3 and Catalase genes delivery *in vivo* through adeno-associated virus**

The full-length FoxO3-TSS mutant (Qi *et al.* 2015) and Catalase coding sequences were cloned into AAV serotype-9 expressing plasmid. AAV9 viruses were packaged and produced using the AAV Helper-Free System (DongBio.Co.Ltd, Shenzhen, China). For AAV9-FoxO3 and AAV9-CAT delivery *in vivo*, viruses were injected via tail vein to C57BL/6 mice at a dose of 1×10^12^ V.G./mouse. AAV9-NC (virus packaged with empty plasmid) served as control. The schematic of AAV9 virus injection can be found in Figure 7A.

**Statistical analysis**

All statistics were calculated using GraphPad Prism 6 Software. All data are presented as the mean ± SEM. Among three or more groups, statistical analysis was performed using one-way ANOVA followed by Dunnett's multiple comparison tests. Comparisons between two groups were analyzed using unpaired Student’s *t*-test. A *p* value of less than 0.05 was considered statistically significant.

**References**

Khan A, Fornes O, Stigliani A, Gheorghe M, Castro-Mondragon JA, van der Lee R, Bessy A, Cheneby J, Kulkarni SR, Tan G, Baranasic D, Arenillas DJ, Sandelin A, Vandepoele K, Lenhard B, Ballester B, Wasserman WW, Parcy F , Mathelier A (2018). JASPAR 2018: update of the open-access database of transcription factor binding profiles and its web framework. *Nucleic Acids Res*. **46**, D260-D266.

Mahmoud AI, Kocabas F, Muralidhar SA, Kimura W, Koura AS, Thet S, Porrello ER , Sadek HA (2013). Meis1 regulates postnatal cardiomyocyte cell cycle arrest. *Nature*. **497**, 249-253.

Qi XF, Chen ZY, Xia JB, Zheng L, Zhao H, Pi LQ, Park KS, Kim SK, Lee KJ , Cai DQ (2015). FoxO3a suppresses the senescence of cardiac microvascular endothelial cells by regulating the ROS-mediated cell cycle. *J Mol Cell Cardiol*. **81**, 114-126.

Qi XF, Zheng L, Lee KJ, Kim DH, Kim CS, Cai DQ, Wu Z, Qin JW, Yu YH , Kim SK (2013). HMG-CoA reductase inhibitors induce apoptosis of lymphoma cells by promoting ROS generation and regulating Akt, Erk and p38 signals via suppression of mevalonate pathway. *Cell Death Dis*. **4**, e518.

Xia JB, Liu GH, Chen ZY, Mao CZ, Zhou DC, Wu HY, Park KS, Zhao H, Kim SK, Cai DQ , Qi XF (2016). Hypoxia/ischemia promotes CXCL10 expression in cardiac microvascular endothelial cells by NFkB activation. *Cytokine*. **81**, 63-70.

Xia JB, Wu HY, Lai BL, Zheng L, Zhou DC, Chang ZS, Mao CZ, Liu GH, Park KS, Zhao H, Kim SK, Song GH, Cai DQ , Qi XF (2017). Gene delivery of hypoxia-inducible VEGF targeting collagen effectively improves cardiac function after myocardial infarction. *Sci Rep*. **7**, 13273.

Zhou DC, Su YH, Jiang FQ, Xia JB, Wu HY, Chang ZS, Peng WT, Song GH, Park KS, Kim SK, Cai DQ, Zheng L , Qi XF (2018). CpG oligodeoxynucleotide preconditioning improves cardiac function after myocardial infarction via modulation of energy metabolism and angiogenesis. *J Cell Physiol*. **233**, 4245-4257.

**Supporting figures and legends**


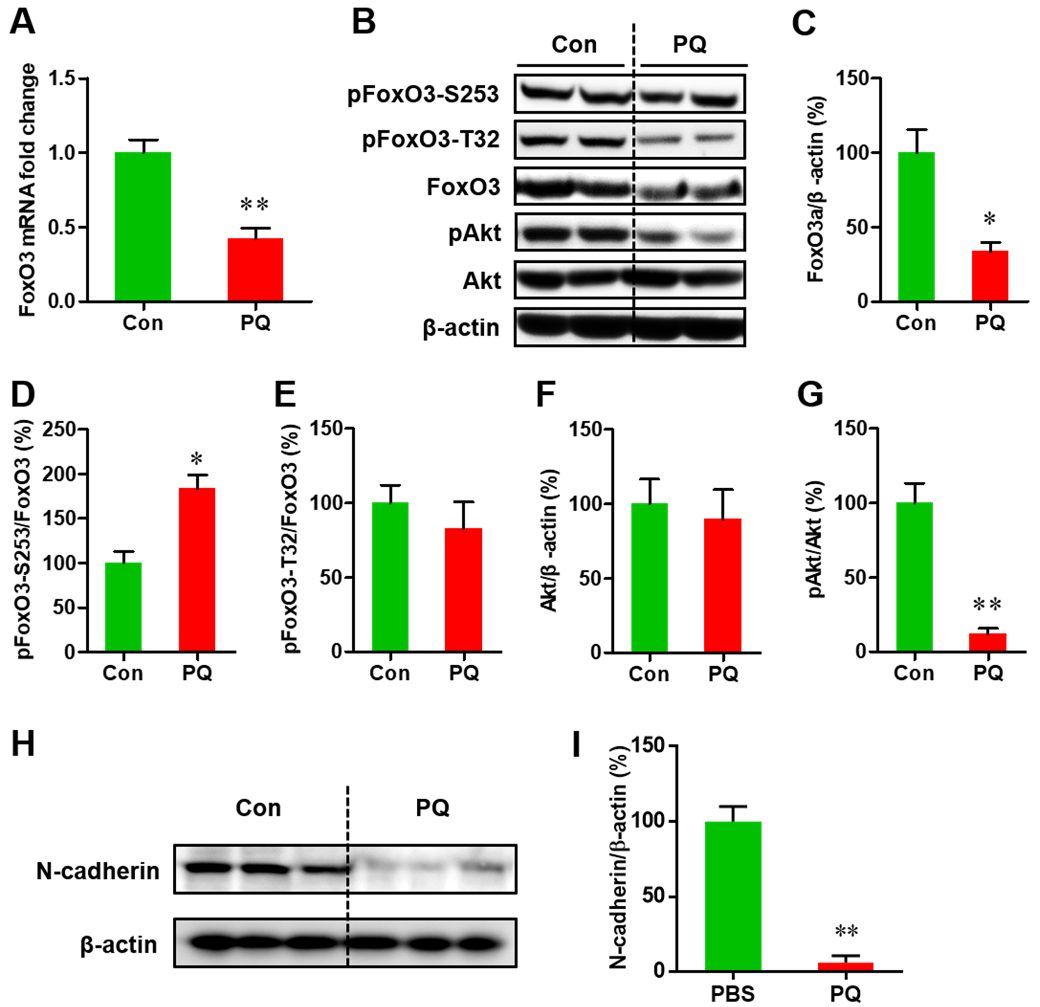


**Figure S1. PQ inhibits the activation of Akt/FoxO3 pathway in H9c2 cells.** H9c2 cells were incubated with PQ (400 μM) for 4 hr, total RNA and protein were then isolated for qRT-PCR and western blotting analysis, respectively. (A) The relative expression of FoxO3 mRNA was shown. Results are presented as mean ± SEM (*n*=3 experiments), ***p*<0.01. (B) The representative images showing protein expression of Akt/FoxO3 pathway with or without phosphorylation. (C-G) The relative expression levels of pan and phosphorylated FoxO3 and Akt were quantified as the percentage of control. Results are presented as mean ± SEM (*n*=3 experiments conducted in duplicate), **p*<0.05, ***p*<0.01 versus control. (H and I) The representative images (H) of N-cadherin protein expression and quantification (I) are shown. Results are presented as mean ± SEM (*n*=3 experiments), ***p*<0.01 versus control.


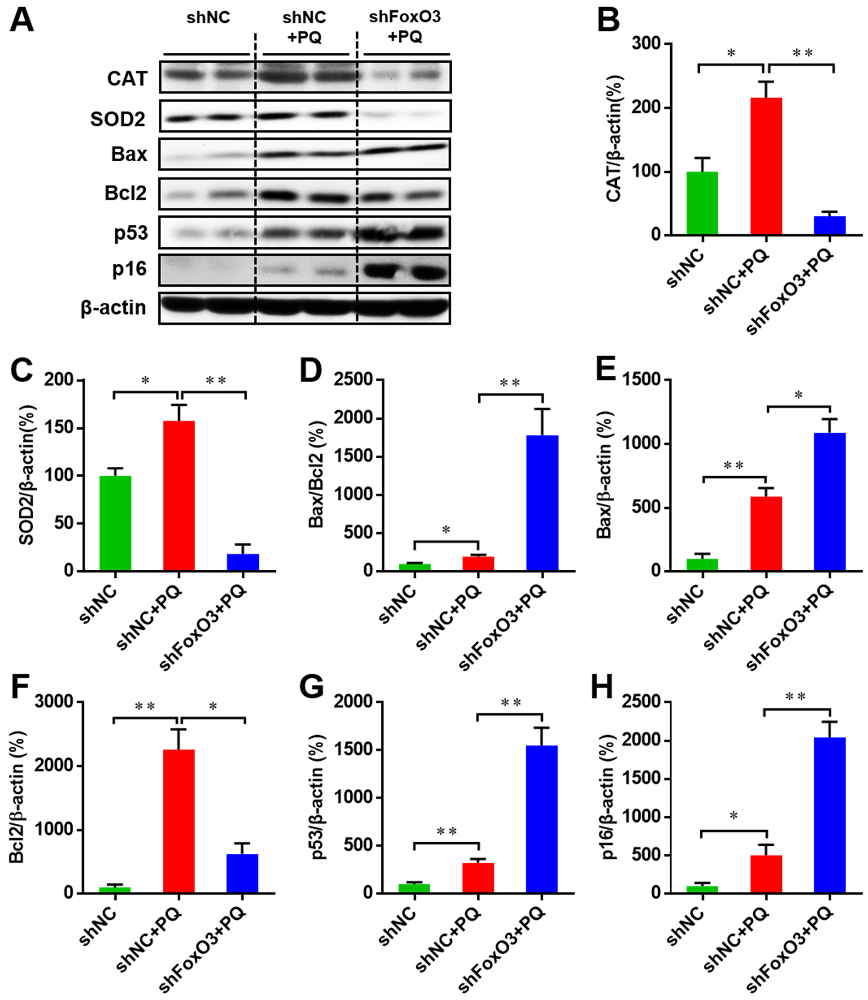


**Figure S2. Effects of FoxO3 silencing on the expression of apoptosis- and oxidative stress-associated proteins in H9c2 cells.** Cells are incubated with or without PQ (400 μM) for 4 hr and subjected to western blotting analysis. (A) Representative images of western blotting for CAT, SOD2, Bax, Bcl2, p53 and p16. (B-H) The relative expression levels of target proteins are quantified as the percentage of control. Results are presented as mean ± SEM (*n*=3 experiments conducted in duplicate), **p*<0.05, ***p*<0.01.


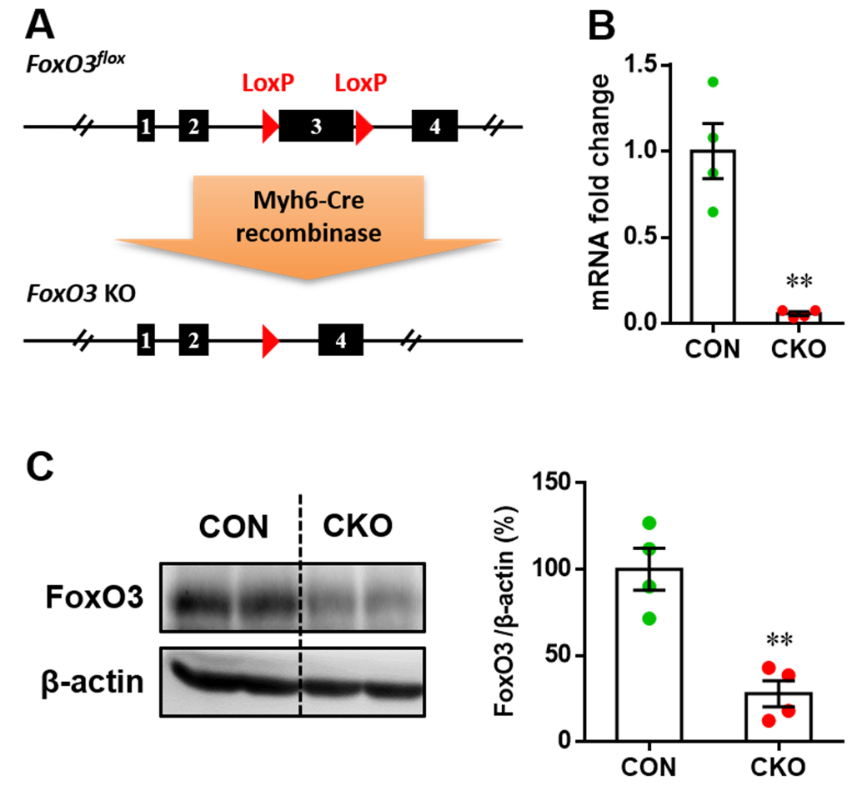


**Figure S3. Generation and verification of FoxO3 cardiac-specific knockout mice.** (A) Schematic of *FoxO3* floxed allele. Control mice were *Myh6-Cre*, *FoxO3* CKO mice were *FoxO3^fl/fl^::Myh6-Cre*. (B) The mRNA expression level of *FoxO3* were determined by qRT-PCR using heart tissues from Control mice and *FoxO3* CKO mice, respectively. Results are presented as mean ± SEM (*n*=4 hearts), ***p*<0.01. (C) The protein expression level of FoxO3 in heart were determined by Western blotting analysis. Representative images (left panel) and quantification analysis (right panel) are shown. Results are presented as mean ± SEM (*n*=4 hearts), ***p*<0.01.


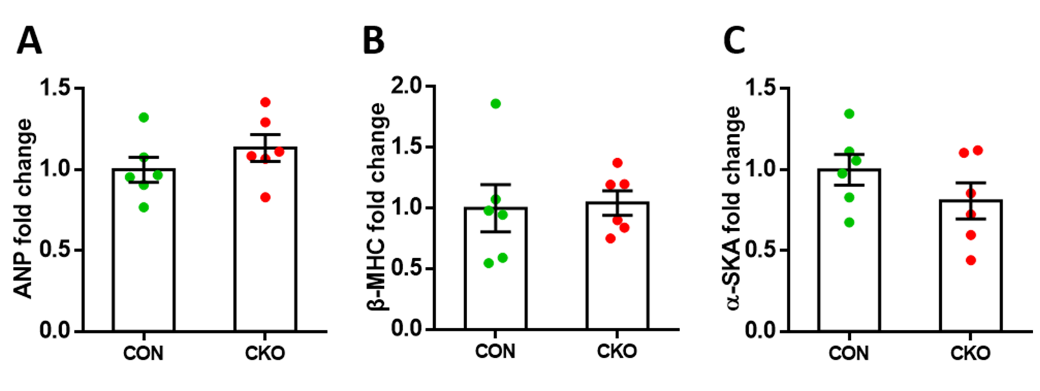


**Figure S4. Expression of cardiac hypertrophy markers.** The mRNA expression of ANP (A), β-MHC (B) and α-SKA (C) in Control and FoxO3 CKO hearts are analyzed by qRT-PCR, respectively. Values are presented as mean ± SEM (*n*=6 hearts per group).


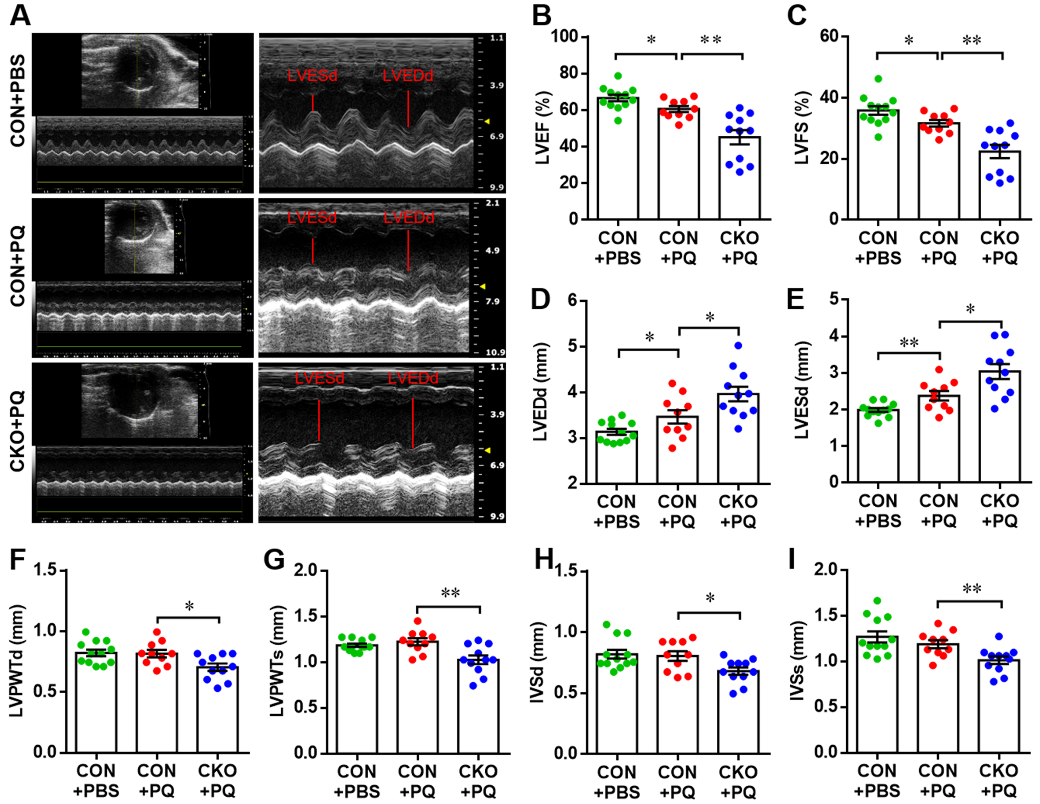


**Figure S5. Cardiac-specific knockout of FoxO3 exacerbates PQ-induced cardiac dysfunction.** Control and FoxO3 CKO mice were injected with PBS or PQ (15 mg/Kg) for four time, followed by echocardiography analysis to evaluate cardiac function. (A) Representative images of M-mode echocardiography showing attenuated cardiac function in CKO mice exposed to PQ compared with CON mice. Right panel, high magnification. (B-I) The left ventricular ejection fraction (LVEF), fractional shortening (LVFS), end-diastolic dimension (LVEDd), end-systolic dimension (LVESd), posterior wall thickness of diastasis (LVPWTd), posterior wall thickness of systole (LVPWTs), interventricular septal end-diastolic thickness (IVSd), and interventricular septal end-systaltic thickness (IVSs) are calculated by M-mode echocardiography in each group. Values are presented as mean ± SEM (*n*=12 hearts for CON+PBS, 10 hearts for CON+PQ, and 11 hearts for CKO+PQ), **p*<0.05, ***p*<0.01.


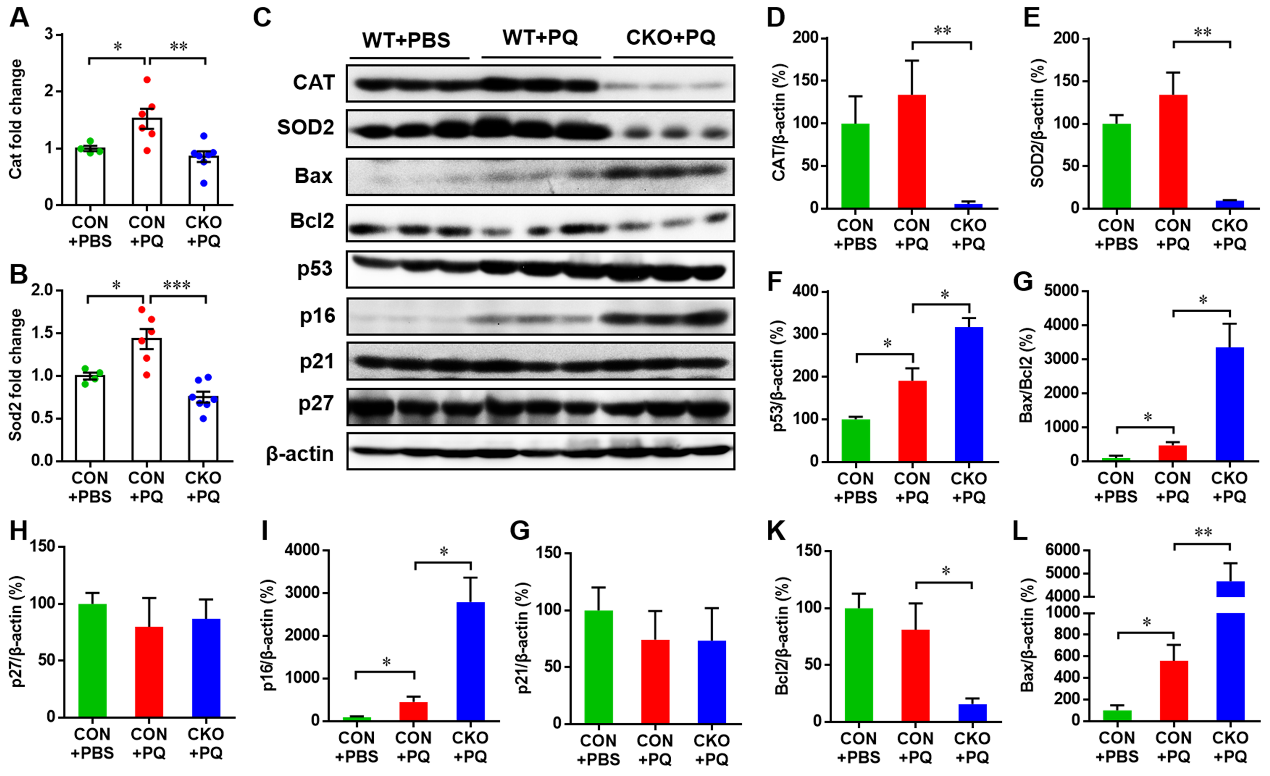


**Figure S6. Effects of FoxO3 knockout on expression of apoptosis- and oxidative stress-associated molecules in heart.** (A and B) The mRNA expression of *Cat* (A) and *Sod2* (B) in hearts are analyzed by qRT-PCR. Values are presented as mean ± SEM (*n*=4 hearts for CON+PBS, 6 hearts for CON+PQ, and 7 hearts for CKO+PQ), **p*<0.05, ***p*<0.01, ****p*<0.001. (C) Representative images of western blotting for CAT, SOD2, Bax, Bcl2, p53, p16, p21, and p27 expression in hearts. β-actin is used as loading control for each sample. (D-L) Relative quantification of protein expression in hearts. The relative expression level of target protein in CON+PBS group is set as 100%. Values are presented as mean ± SEM (*n*=3 hearts for each group), **p*<0.05, ***p*<0.01.


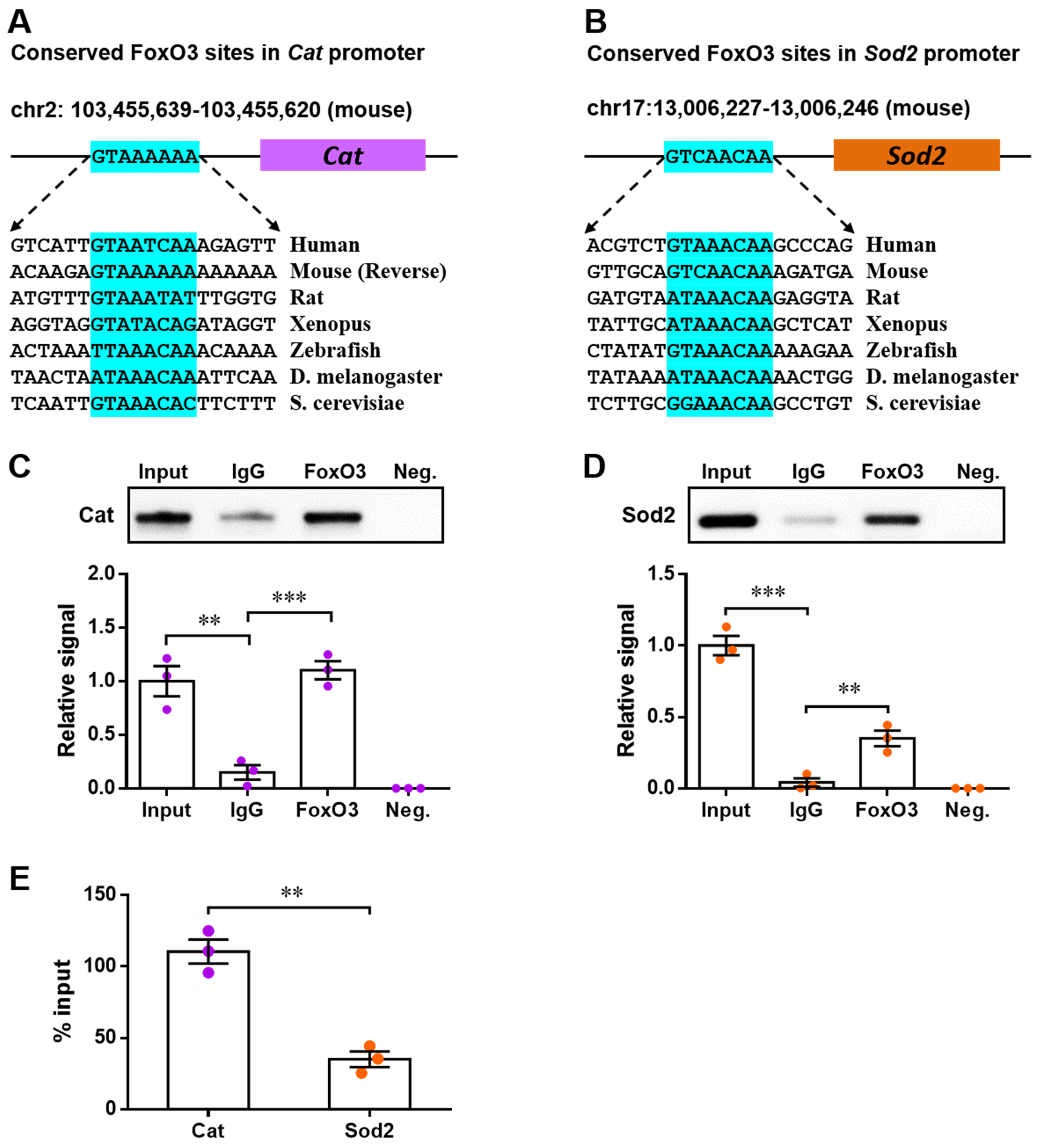


**Figure S7. *In vivo* interactions of FoxO3 with *Cat* and *Sod2* promoters in mice heart.** (A and B) Highly conserved FoxO3 motifs located in *Cat* (A) and *Sod2* (B) promoters for different species. Among the JASPAR-predicted FoxO3 motifs in promoter (-2 kb upstream of TSS), only one with highest score for each species are selected and aligned. (C and D) Up panels, representative images of ChIP assay confirm the *in vivo* interaction of FoxO3 with *Cat* (C) or *Sod2* (D) promoters. Low panel, relative quantification of the binding ability of FoxO3 with *Cat* (C) or *Sod2* (D) promoters. DNA signals for input are set as 1 (100%). Neg., negative control without DNA template. Values are presented as mean ± SEM (*n*=3 hearts), ***p*<0.01, ****p*<0.001. (E) Cat and Sod2 promoter DNA immunoprecipitated by FoxO3 protein are compared. FoxO3-immunoprecipitated DNA is normalized to input. Values are presented as mean ± SEM (*n*=3 hearts), ***p*<0.01 versus control.


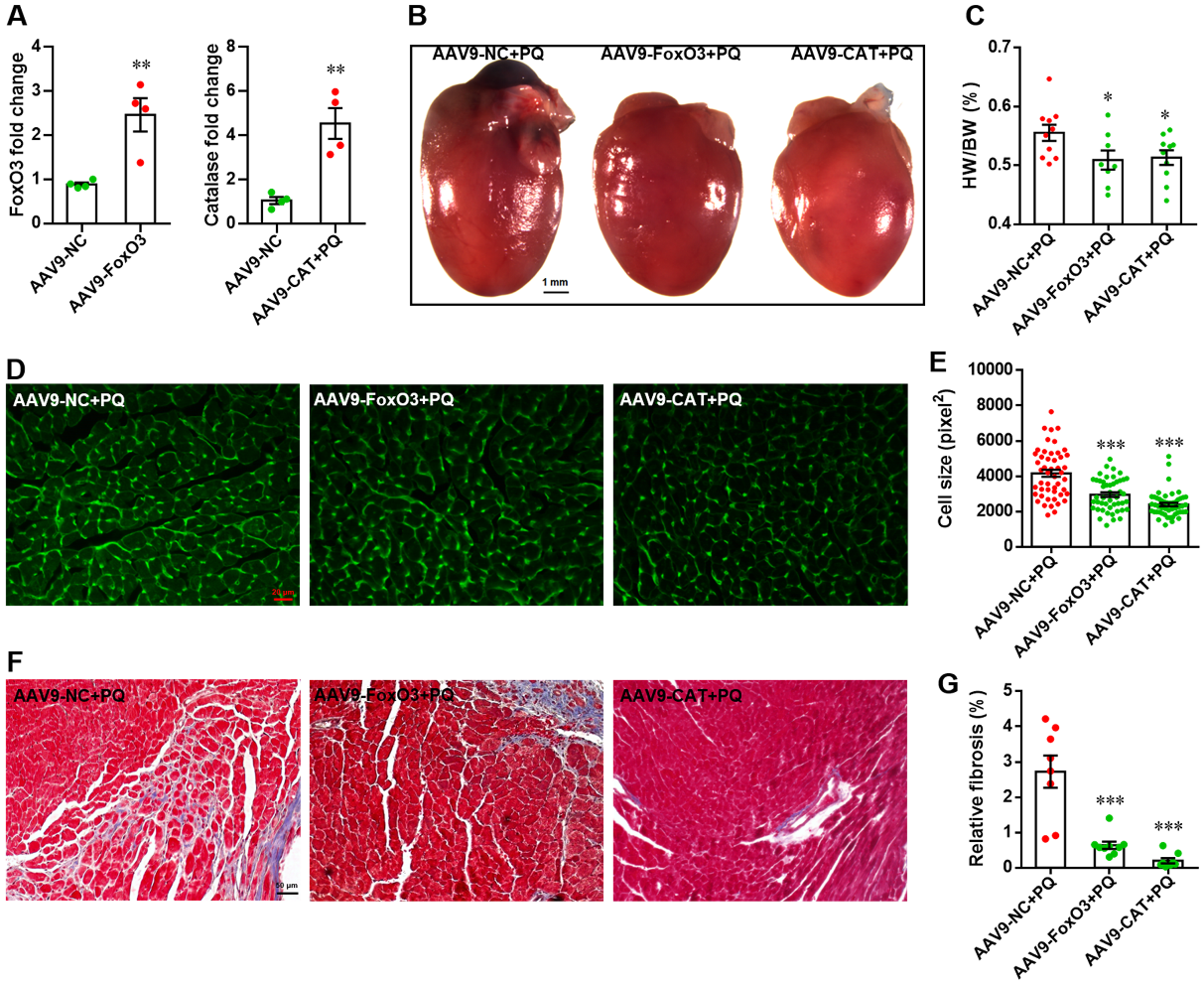


**Figure S8. Overexpression of FoxO3 and CAT attenuate PQ-induced cardiac remodeling.** (A) Overexpression of FoxO3 (left) and CAT (right) in hearts were confirmed by qRT-PCR analysis. Results are presented as mean ± SEM (*n*=4 hearts), ***p*<0.001 versus control. (B) Representative images of hearts indicate FoxO3 and CAT attenuate PQ-induced cardiac hypertrophy. (C) The ratio of heart weight to body weight (HW/BW) is analyzed. Results are presented as mean ± SEM (*n*=8~10 heart per group), **p*<0.05 versus control. (D) Representative images of WGA staining for each condition are shown. (E) Cell size of cardiomyocytes is quantified by Image-Pro Plus version 6.0 software. Results are presented as mean ± SEM (*n*=40 sections from 8 hearts per group), ****p*<0.001 versus control. (F) Representative images of heart section with Masson’s trichrome staining. (G) Relative cardiac fibrosis is quantified and expressed as the percentage of whole ventricle. Results are presented as mean ± SEM (*n*=8 hearts per group), ****p*<0.001 versus control.

**Supporting tables**

**Supporting table 1. FoxO3 binding sites in the promoter regions of mice Cat.**

| **Matrix ID** | **Name** | **Score** | **Relative score** | **Start** | **End** | **Predicted sequence** |
| --- | --- | --- | --- | --- | --- | --- |
| MA0157.2 | FOXO3 | 9.83891 | 0.905451 | 871 | 878 | GTAAAAAA |
| MA0157.1 | FOXO3 | 9.37963 | 0.907206 | 1187 | 1194 | TGTAAATA |
| MA0157.1 | FOXO3 | 8.30279 | 0.871033 | 1648 | 1655 | TGTAAAAA |
| MA0157.1 | FOXO3 | 8.279 | 0.870233 | 1646 | 1653 | TAAAAACA |
| MA0157.2 | FOXO3 | 8.05606 | 0.869495 | 1186 | 1193 | GTAAATAT |
| MA0157.2 | FOXO3 | 8.00767 | 0.868519 | 1730 | 1737 | GCAAACAA |
| MA0157.2 | FOXO3 | 8.00767 | 0.868519 | 1739 | 1746 | GTAAGCAA |
| MA0157.1 | FOXO3 | 7.70982 | 0.851113 | 1666 | 1673 | GGTATACA |
| MA0157.2 | FOXO3 | 7.4599 | 0.857472 | 1059 | 1066 | TTAAACAT |
| MA0157.2 | FOXO3 | 7.25641 | 0.853368 | 1371 | 1378 | ATAAACAG |
| MA0157.1 | FOXO3 | 6.85145 | 0.822279 | 887 | 894 | TGAGAACA |
| MA0157.1 | FOXO3 | 6.60919 | 0.814141 | 1207 | 1214 | GTAAAACA |
| MA0157.1 | FOXO3 | 6.31883 | 0.804387 | 1372 | 1379 | CATAAACA |
| MA0157.1 | FOXO3 | 6.28539 | 0.803264 | 884 | 891 | TCTCAACA |
| MA0157.1 | FOXO3 | 6.26726 | 0.802655 | 1091 | 1098 | GATACACA |
| MA0157.1 | FOXO3 | 6.25847 | 0.80236 | 1321 | 1328 | GGAAACCA |
| MA0157.2 | FOXO3 | 5.66303 | 0.821234 | 1647 | 1654 | GTAAAAAC |
| MA0157.2 | FOXO3 | 5.0605 | 0.809082 | 1667 | 1674 | GTATACAG |
| MA0157.2 | FOXO3 | 4.96759 | 0.807208 | 6 | 13 | ATAAAAAT |
| MA0157.2 | FOXO3 | 4.8436 | 0.804708 | 1207 | 1214 | GTAAAACA |
| MA0157.2 | FOXO3 | 4.69821 | 0.801776 | 1321 | 1328 | GGAAACCA |

The promoters (-2,000 to -1, upstream of TSS) of target gene were predicted by JASPAR 2018 online software. The binding site with highest score (red) was further analyzed by luciferase report gene system.

**Supporting table 2. FoxO3 binding sites in the promoter regions of mice Sod2.**

| **Matrix ID** | **Name** | **Score** | **Relative score** | **Start** | **End** | **Predicted sequence** |
| --- | --- | --- | --- | --- | --- | --- |
| MA0157.2 | FOXO3 | 9.90645 | 0.906813 | 395 | 402 | GTCAACAA |
| MA0157.1 | FOXO3 | 9.76145 | 0.920032 | 1445 | 1452 | TGGAAACA |
| MA0157.2 | FOXO3 | 9.69352 | 0.902518 | 1493 | 1500 | GGAAACAA |
| MA0157.1 | FOXO3 | 9.16847 | 0.900112 | 1492 | 1499 | GGGAAACA |
| MA0157.2 | FOXO3 | 9.14575 | 0.891471 | 1446 | 1453 | GGAAACAT |
| MA0157.1 | FOXO3 | 8.78665 | 0.887286 | 467 | 474 | GGTAAACT |
| MA0157.1 | FOXO3 | 8.19509 | 0.867415 | 1181 | 1188 | TGTGAACA |
| MA0157.1 | FOXO3 | 8.03598 | 0.86207 | 1661 | 1668 | TGAACACA |
| MA0157.1 | FOXO3 | 7.49457 | 0.843883 | 1383 | 1390 | AGAAAACA |
| MA0157.2 | FOXO3 | 7.4599 | 0.857472 | 799 | 806 | TTAAACAT |
| MA0157.1 | FOXO3 | 6.36617 | 0.805978 | 1334 | 1341 | GGAAAAAA |
| MA0157.1 | FOXO3 | 6.36617 | 0.805978 | 1397 | 1404 | GGAAGACA |
| MA0157.1 | FOXO3 | 6.28539 | 0.803264 | 562 | 569 | TCTCAACA |
| MA0157.1 | FOXO3 | 6.28539 | 0.803264 | 1388 | 1395 | TCTAATCA |
| MA0157.1 | FOXO3 | 6.25847 | 0.80236 | 479 | 486 | GGAAACCA |
| MA0157.1 | FOXO3 | 6.25847 | 0.80236 | 681 | 688 | GGAAATCA |
| MA0157.1 | FOXO3 | 6.25847 | 0.80236 | 1265 | 1272 | GGAAAGCA |
| MA0157.2 | FOXO3 | 5.95021 | 0.827025 | 1180 | 1187 | GTGAACAG |
| MA0157.2 | FOXO3 | 5.51536 | 0.818256 | 1149 | 1156 | ATAAAAAA |
| MA0157.2 | FOXO3 | 5.07284 | 0.809331 | 1650 | 1657 | GGCAACAA |
| MA0157.2 | FOXO3 | 5.0605 | 0.809082 | 1752 | 1759 | GTAATCAG |
| MA0157.2 | FOXO3 | 5.0053 | 0.807969 | 1334 | 1341 | GGAAAAAA |
| MA0157.2 | FOXO3 | 4.69821 | 0.801776 | 479 | 486 | GGAAACCA |

The promoters (-2,000 to -1, upstream of TSS) of target gene were predicted by JASPAR 2018 online software. The binding site with highest score (red) was further analyzed by luciferase report gene system.
